# Cost-Effectiveness of Treatment Decisions for Early Childhood Caries in Infants and Toddlers: A Systematic Review

**DOI:** 10.3390/medicina59101865

**Published:** 2023-10-20

**Authors:** Thomas Gerhard Wolf, Guglielmo Campus

**Affiliations:** 1 Department of Restorative, Preventive and Pediatric Dentistry, University of Bern, Freiburgstrasse 7, 3010 Bern, Switzerland; 2 Department of Periodontology and Operative Dentistry, University Medical Center of the Johannes Gutenberg University Mainz, 55116 Mainz, Germany; 3 Department of Surgery, Microsurgery and Medicine Sciences, School of Dentistry, University of Sassari, Viale San Pietro, 07100 Sassari, Italy; guglielmo.campus@unibe.ch

**Keywords:** cost-effectiveness, early childhood caries, infant, treatment, toddler, young children

## Abstract

*Background and Objectives:* Early childhood caries (ECC) is a multifactorial, biofilm-mediated, sugar-related, dynamic disease of primary dental hard tissues occurring in varying degrees of severity in infants and toddlers. Untreated ECC may lead to pain, infections, and severe systemic complications. The aim of this study was to systematically review and evaluate the scientific evidence on the cost-effectiveness of treatment decisions in ECC in infants and toddlers. *Materials and Methods:* Observational epidemiological studies, i.e., cohort studies, case–control studies, and randomized controlled trials, reporting cost-effectiveness of treatment decisions in ECC in infants and toddlers were included in the systematic review following the PRISMA guidelines. Using an ad hoc search with search terms or keywords (MeSH), electronic databases Embase, MEDLINE via PubMed, Scopus, and gray literature were searched. *Results:* The search identified 494 articles, of which 446 remained after removing duplicates. A total of 417 articles were excluded after title and abstract evaluation; 29 full-text articles were screened for eligibility, and five articles were discarded. Twenty-four full-text articles were included in the systematic review, assigning 17 to prevention and seven to restoration. Results were heterogeneous; comparability of included studies is difficult because of the different methodologies used. Conflicting efficacies were demonstrated for different interventions implemented, and cost-effectiveness data were documented. *Conclusions:* Socioeconomic, cultural, and ethnic differences must be considered when comparing conditions in terms of cost-effectiveness. A paradigm shift from surgical towards preventive treatment decisions can be observed. Cost-effectiveness studies on therapies for ECC in infants and toddlers are needed to identify the best practice approach and the most cost-effective therapy decisions.

## 1. Introduction

Early childhood caries (ECC) has gained importance in the past decade worldwide [1,2,3,4,5,6]. ECC, in the past also occasionally called as “baby bottle syndrome”, “breastfeeding caries”, “bottle mouth caries”, “rampant caries”, or “wild caries”, is characterized by carious lesions that produce an invasion of the dental hard tissue on deciduous tooth surfaces and occur within the first three years of life in infants and toddlers [1,2,3,4,5,6]. Dental caries remains the most common disease of mankind and plays an important role in ensuring good oral and general health [6]. Early childhood caries can be differentiated into three levels of severity, with the mild/moderate form often occurring between the ages of 2 and 5 years, where it is also stated to occur up to 71 months, which corresponds to under 6 years [1,2,3,4,5,6,7]. The mild/moderate form often occurs between the ages of 2 and 5 years on deciduous molars and/or incisors (ECC type I), the moderate to severe form occurs on maxillary (maxillary) incisors with caries-free mandibular (mandibular) incisors often shortly after deciduous tooth eruption (ECC type II), and the severe form involves almost all deciduous teeth including the mandibular incisors in predominantly 3- to 5-year-old infants (EDD type III) [1]. Prevalence is reported between 5% and 20% in Germany [8,9,10] and between 3% and 45% worldwide [11] in epidemiological studies. In the past, early childhood caries could only be diagnosed with the dental screening examination for children beginning at 30 months of age; however, between 7% and 20% of children already have early childhood caries at this time [12]. An association of population strata with both low social status and low education levels can be observed, with 2% of children already showing 52% of caries [13]. However, middle class populations were also clustered in early childhood caries due to misinformation about causes of the disease [14].

The causes for the occurrence of early childhood caries are manifold. These include both behavioral and social risk factors [15]. In addition to a possible transmission of cariogenic microbes from mother to child, e.g., by licking the bottle or the pacifier, the consumption of sugary and acidic foods or frequent snacks such as the consumption of sweets are also significant factors. In addition to nocturne bottle sucking, inadequate oral and dental hygiene as well as irregular intake of fluoridation measures for prophylaxis are also significant for the development and progression of the disease [15].

The possible consequences of early childhood caries are pain and multiple inflammations with an early loss of milk teeth and thus resulting in both aesthetic and functional impairment [2,3,4,5,6]. However, it can also lead to possible damage to the germs of permanent teeth, which is often accompanied by impaired tooth eruption, developmental disorders of the upper and lower jaw, impaired speech development, insufficient nutritional behavior, and dysfunctional chewing or swallowing ability [2,3,4,5,6]. In addition to the psychosocial developmental deficits caused by the caries that have occurred or the loss of teeth, especially in the visible anterior region, a lack of compliance for future visits to the dentist often develops [15]. While mild to moderate early childhood caries (ECC type I–II) is often still feasible with premedication (midazolam), nitrous oxide, or behavioral guidance/hypnosis, in the case of severe early childhood caries (ECC type III), often only remediation under ITN (intubation anesthesia, general anesthesia) remains due to the intensive and time-consuming treatment with simultaneous frequent lack of compliance [2,3,4,5,6]. However, the dangers of both sedation and general anesthesia due to dental caries disease must not be underestimated under any circumstances, because such sedative/anesthetic treatments pose an enormous health risk for an infant or young child and not infrequently lead to complications and even death [16,17].

Intervention strategies with in-depth diagnostics and preventive as well as therapeutic measures are necessary [18,19,20]. Above all, prevention in combination with an interdisciplinary approach should enable dentists to work better with family doctors, pediatricians, gynecologists, as well as midwives in this area to prevent caries as early as possible. In order to detect and diagnose the disease even before group or individual prophylaxis is reached at preschool age, the benefits catalog of the statutory health insurance in Germany was expanded about four years ago to include three additional dental screening examinations for toddlers from 6 months to 33 months of age [15]. The early detection examinations are to take place between the 6th and 9th (FU1), the 10th and 20th (FU2), and from the 21st month of life (FU3). The dental screening examinations focus on the causal development of caries and therefore include, in addition to a detailed examination of the child, counseling of the parents with specific instructions for regular, daily tooth brushing and enamel hardening with fluoride varnish twice per calendar half-year, to which toddlers are legally entitled.

Cost efficiency has become an enormously important aspect of public health care. Cost-effectiveness analysis is defined as “a way to examine both the costs and health outcomes of one or more interventions. It compares an intervention to another intervention (or the status quo) by estimating how much it costs to gain a unit of a health outcome, like a life year gained or a death prevented” [21]. By means of cost-effectiveness analyses of different therapies, additional information can be obtained in the sense of the cost-effectiveness requirement of the statutory or state health insurance system, in which every medical treatment must be sufficient, appropriate, and economical, which can influence participative decision-making between dentist and patient (or child accompanied by parents). To date, few systematic reviews have focused on cost-effectiveness in children. To the best of our knowledge, no systematic review for cost-effectiveness of treatment decisions for ECC in infants and young children has been conducted.

Therefore, the aim of this study is to systematically review and summarize research findings on the cost-effectiveness of treatment decisions for early childhood caries in infants and toddlers.

## 2. Materials and Methods

### 2.1. Protocol and Registration

This systematic review was registered with the International Center for Open Science (Center for Open Science, 210 Ridge McIntire Road, Suite 500, Charlottesville, VA, USA) and addresses the cost-effectiveness of treatment decisions of early childhood caries in infants and toddlers and includes in vivo studies from birth to 71 months [22]. Data collected are in accordance with the most recent Preferred Reporting Items for Systematic Reviews and Meta-Analyses Statement (PRISMA 2020) guidelines for reporting systematic reviews of health care interventions [23].

### 2.2. Inclusion and Exclusion Criteria

The inclusion criteria were as follows: observational epidemiologic studies (cohort studies, case–control studies, and randomized controlled trials) that reported on the cost-effectiveness of treatment decisions in early childhood caries in infants and toddlers. Studies that evaluated cost-effectiveness using cost-effectiveness modeling (e.g., Markov model) or that only performed cost simulation without the direct link to clinical intervention were explicitly excluded.

The following PICO questions (population, intervention, comparison, and outcome) were formulated [24]:

- P = infants and toddlers aged 0 to 71 months;
- I = treatments for early childhood caries (ECC);
- C = cost-effectiveness of treatment decisions;
- O = summary or relative order/priority of the strategies.

Due to the great heterogeneity in the study and the types of dental materials used in this research area, no common comparator was defined for the included studies. Nevertheless, all studies were included and comparatively evaluated regarding the outcome “cost-effectiveness of treatment decisions”.

The SPIDER (sample, phenomenon of interest, design, evaluation, research) method was also used to create the search strategy [25,26]. Thus, the following conceptualizations were included in conjunction with the concept of “Therapeutic Decision Making”:

- S = population*, subject*, high risk*, group*, age group*.
- PI = The terms “decision making, cost, cost-effec*, beneficial, prevention, treatment, caries, dental caries” are used to evaluate the concept of “treatment decision making”.
- D = Quantitative original publications are considered.
- E = The search strategy is not restricted regarding the evaluation (E) of the publication since there are probably few comprehensive data on this area.
- R = RCT, cohort studies, and case–control studies are the studies of first choice. No time restriction is applied as an exclusion criterion. Publications in English, German, French, or Italian will be considered.

### 2.3. Data Sources, Search Strategy and Study Selection

Detailed search terms and a search strategy were created using Boolean operators. An ad hoc literature search was performed using search terms, keywords (MeSH—medical subject headings), in the electronic databases MEDLINE via PubMed, Embase, Scopus, and Open Grey Literature (https://www.opengrey.eu, accessed on 14 July 2023). The last search for all electronic databases took place on 14 July 2023. In addition, a hand search was performed. Articles in English, German, French, or Italian were considered. Furthermore, the references of all the included studies were also hand-searched. The electronic databases were searched according to their advanced search syntax using a search strategy identical for the three databases. Duplicates were removed from the literature search results. Cross-references were also made using the bibliographies of the full-text articles. Data were reported according to the updated Preferred Reporting Items for Systematic Reviews and Meta-Analyses 2020 (PRISMA) guidelines [23]. The PRISMA checklist is available in the Supplementary Material (Figure S1). The search strategy included search terms for each selected electronic database. For MEDLINE via Pubmed, the following search terms were used: ((“carie” [All Fields] OR “dental caries” [MeSH Terms] OR (“dental” [All Fields] AND “caries” [All Fields]) OR “dental caries” [All Fields] OR “caries” [All Fields]) AND ((“manage” [All Fields] OR “managed” [All Fields] OR “management s” [All Fields] OR “managements” [All Fields] OR “manager” [All Fields] OR “manager s” [All Fields] OR “managers” [All Fields] OR “manages” [All Fields] OR “managing” [All Fields] OR “managment” [All Fields] OR “organization and administration” [MeSH Terms] OR (“organization” [All Fields] AND “administration” [All Fields]) OR “organization and administration” [All Fields] OR “management” [All Fields] OR “disease management” [MeSH Terms] OR (“disease” [All Fields] AND “management” [All Fields]) OR “disease management” [All Fields]) AND (“infant” [MeSH Terms] OR “infant” [MeSH Terms:noexp] OR “child, preschool” [MeSH Terms] OR “child” [MeSH Terms:noexp])) AND (“infant” [MeSH Terms] OR “infant” [MeSH Terms:noexp] OR “child, preschool” [MeSH Terms] OR “child” [MeSH Terms:noexp]) AND ((((“carie” [All Fields] OR “dental caries” [MeSH Terms] OR (“dental” [All Fields] AND “caries” [All Fields]) OR “dental caries” [All Fields] OR “caries” [All Fields]) AND (“clinical trial” [Publication Type] OR “randomized controlled trial” [Publication Type]) AND (“infant” [MeSH Terms] OR “child” [MeSH Terms:noexp] OR “infant” [MeSH Terms:noexp] OR “child, preschool” [MeSH Terms])) OR (“early” [All Fields] AND (“childhood” [All Fields] OR “childhoods” [All Fields]) AND (“carie” [All Fields] OR “dental caries” [MeSH Terms] OR (“dental” [All Fields] AND “caries” [All Fields]) OR “dental caries” [All Fields] OR “caries” [All Fields]))) AND (“infant” [MeSH Terms] OR “infant” [MeSH Terms:noexp] OR “child, preschool” [MeSH Terms] OR “child” [MeSH Terms:noexp])) AND (“infant” [MeSH Terms] OR “infant” [MeSH Terms:noexp] OR “child, preschool” [MeSH Terms] OR “child” [MeSH Terms:noexp]) AND ((“cost benefit analysis” [MeSH Terms] OR (“cost benefit” [All Fields] AND “analysis” [All Fields]) OR “cost benefit analysis” [All Fields] OR (“economic” [All Fields] AND “evaluation” [All Fields]) OR “economic evaluation” [All Fields]) AND (“infant” [MeSH Terms] OR “infant” [MeSH Terms:noexp] OR “child, preschool” [MeSH Terms] OR “child” [MeSH Terms:noexp]))) AND (allinfant [Filter] OR infant [Filter] OR preschoolchild [Filter] OR child [Filter]).

The titles and abstracts of all the identified studies were first reviewed independently by the authors. Abstracts were assessed if the title indicated possible inclusion. After careful review of the abstracts, those manuscripts that were eligible for review and available in full text were selected. Full texts were then assessed and reviewed independently for content and inclusion in the study. In case of disagreement, there was an opportunity for agreement on inclusion of studies or data extraction by consulting with an additional researcher. Microsoft Excel spreadsheets (Microsoft Corporation, Redmont, WA, USA) were used for recording and data extraction. They were completed so that each eligible study was assigned a study ID that included the author’s name, year of publication, and country of study conduct.

The following data were also extracted:

- Setting: duration of study, site of data collection or examination or treatment, number of patients, age of patients, type of treatment, type of control group, number of teeth or lesions, if any, follow-up, cost of treatment, cost of control group, clinical outcome;
- Materials: prevention or restoration (classification, name);
- Methodological aspects: study design, evaluation criteria, for e.g., restoration survival or intervention.

### 2.4. Data Collection and Evaluation of Study Quality

Data collection and synthesis were performed using an ad hoc designed extraction table without masking the name of the journal, title, or even authors. Studies were classified chronologically according to their country of study conduct, number of study participants, location (e.g., practice/clinic) of study conduct, age of study participants, and study quality assessment. Furthermore, included studies were classified into two main groups of prevention according to their primary outcome [18,27,28,29,30,31,32,33,34,35,36,37,38,39,40,41] and restoration [42,43,44,45,46,47,48] with different subgroups divided at prevention (outreach/education/education, sealing, fluoride varnish, and silver diamine fluoride (SDF) and atraumatic restorative treatment (ART) as well as restoration (filling and crown). For ease of synthesis and clarity, the results were summarized in tables. Accordingly, for each article, these data were queried and recorded when available: (a) authors, year of publication, source of publication, site of study conduct, duration of study in months, site of study conduct (practice/clinic), number of participants, age of participants in years, and quality assessment (risk of bias) of studies, (b) study design and treatment (intervention) and control groups, and (c) cost of treatment (intervention), cost of control group, and clinical outcome. Areas (b) and (c) were respectively divided into prevention (information/education/education, sealing, fluoride varnish, and silver diamine fluoride (SDF) and atraumatic restorative treatment (ART)) and restoration (filling and crown).

An individual quality assessment tool developed by the National Heart, Lung, and Blood Institute of the National Institute of Health (Bethesda, Maryland, USA) for observational cohorts, case–control study, and controlled intervention studies was used (Study Quality Assessment Tools, NHLBI, NHI; https://www.nhlbi.nih.gov/health-topics/study-quality-assessment-tools, last accessed 12 September 2023). The quality of the studies was assessed according to the following criteria: low/poor quality (“poor”): 0–6, adequate quality (“fair”): 7–11, and good quality (“good”). The quality assessment of the studies is intended to provide information on the internal validity of a study and to consider the risk of bias through critical evaluation.

## 3. Results

The search identified a total of 494 articles; 446 were selected after duplicates were removed and 417 articles were excluded after review or evaluation of title and abstract. The list of excluded studies after initial review can be found in Supplementary Materials (Table S1). Twenty-nine articles were reviewed for eligibility by full text, of which five articles were discarded after review of the full text. No articles were added by hand search, i.e., without Boolean search terms via electronic databases. There were 24 full-text articles included in the review work, of which 17 articles were assigned to prevention and 7 to restoration. The flowchart of the search can be seen in Supplementary Materials (Figure S2).

There were ultimately 24 articles included in this systematic review, with two articles each reporting the same study [34,49] as well as [34,48]. While the articles [34,49] were about similar results, the articles [43,48] represented the baseline study [43] and the follow-up study with final clinical trial results [48]. Of the 24 articles included in the systematic review, 17 articles could be classified as prevention [18,27,28,29,30,31,32,33,34,35,36,37,38,39,40,41,49]. Seven studies were classified as restoration [42,43,44,45,46,47,48]. All included studies were published in the last decade [18,27,28,29,30,31,32,33,34,35,36,37,38,39,40,41,42,43,44,45,46,47,48,49]. Most of the included articles (12 studies) and thus half of the articles were published in the last 3 years from 2020 to 2023 [18,33,34,35,36,37,38,39,40,41,48,49].

### Study Characteristics

The included studies were a total of seven retrospective cohort studies [27,31,33,37,39,41,42], one uncontrolled [29], one cohort study [23], and randomized controlled trials [18,28,30,32,34,35,36,38,40,43,44,45,46,47,48,49] (Table 1). The summaries of the selected studies are presented in Table 2. Most studies were conducted in the United Kingdom of Great Britain and Northern Ireland [28,29,31,34,35,47,49], the United States [27,28,37,42], and Brazil [32,43,46,48]. Sixteen studies were conducted in public health facilities or schools [18,27,28,29,30,31,32,35,36,38,40,41,43,44,48,49], one study in a specialized dental practice [42], one study in a day care center [46], four studies in general or private dental practices [33,34,45,47], one study in an outpatient clinic or operating room [37], and one study that examined dental services only [46]. Of the included studies, a total of 14 were considered “good” [27,28,31,32,35,36,40,41,43,44,45,46,47,48] and ten studies rated as “fair” [18,29,30,33,34,37,38,39,42,49] (Table 2 and Table 3).

In the prevention domain, there were one study each on outreach/education and sealant, eight studies on fluoride varnish [18,28,29,33,35,38,41,49], and seven studies on silver diamine fluoride (SDF) and atraumatic restorative treatment (ART) [30,31,32,33,36,39,40]. In the restorative field, three studies were on filling [43,46,48] and four on crown [42,44,45,47]. Due to the heterogeneity in both the methodology used on treatment and control groups and the different clinical outcomes, a further synthesis of the results on cost-effectiveness (Table 4) of individual techniques or methods is not possible. In total, the 24 articles included 77,546 children in the systematic review. Eighteen studies had a sample size of more than 100 participants [18,27,28,29,30,31,33,34,35,37,38,39,41,43,44,46,47,48]. Most studies have been conducted in children between the ages of 3 and 7 years.

## 4. Discussion

### 4.1. Aim of the Study and Main Findings

The aim of this study was to systematically review and summarize current research on the cost-effectiveness of treatment decisions for early childhood caries (ECC) in infants and toddlers. Using ad hoc search terms (MeSH), keywords, and a defined search strategy, the electronic databases Scopus, MEDLINE via PubMed, Embase, and gray literature were searched. Observational epidemiologic studies, i.e., cohort studies, case–control studies, and randomized controlled trials that reported on the cost-effectiveness of treatment decisions of early childhood caries in infants and toddlers, according to the PRISMA guidelines, were included in the study. Cost-effectiveness modeling (e.g., Markov model) or cost simulation without direct link to clinical intervention were explicitly excluded. This systematic review is one of the very few systematic reviews focusing on cost-effectiveness in children [50]. However, to the best of our knowledge, no systematic review of treatment decisions for ECC in infants and toddlers has been conducted so far. A total of 494 articles were identified, and after title and abstract evaluation, hand searching, and full-text review, 24 full-text articles were finally included in the systematic review [12,20,21,22,23,24,25,26,27,28,29,30,31,32,33,34,35,36,37,38,39,40,41,42], with 17 articles classified as prevention [18,27,28,29,30,31,32,33,34,35,36,37,38,39,40,41,49] and seven articles classified as restoration [35,36,37,38,39,40,41]. Dental caries remains the most common disease in humans [51,52], and early childhood caries in infants and toddlers from birth to approximately age seven can have serious health and economic consequences [2,3,9]. Non-treatment can lead to serious infections, developmental disorders, and massive losses in quality of life as well as financial burdens [8,9,15]. Depending on the severity, treatment is often only possible with sedation measures or even general anesthesia [8,16].

### 4.2. Classification of Studies

Although the included studies could be classified/assigned into the two areas of prevention and restoration [18,27,28,29,30,31,32,33,34,35,36,37,38,39,40,41,42,43,44,45,46,47,48,49], each with subgroups on (1) prevention: education/outreach, sealing, fluoride varnish, and silver diamine fluoride (SDF) and atraumatic restorative treatment (ART) [18,27,28,29,30,31,32,33,34,35,36,37,38,39,40,41,49] and on (2) restoration with filling and crown restoration [35,36,37,38,39,40,41], a concrete comparison was very difficult due to the lack of homogeneity in the results. Direct comparisons between the groups could be drawn neither related to the therapeutic intervention nor to the methodology used to measure cost-effectiveness. However, due to the given heterogeneity in the results, neither a further synthesis nor a meta-analysis could be performed. In addition to the limitation that the systematic review included too few studies or that no more literature was available in the electronic databases without a time limit, the prevailing socioeconomic, cultural, and ethnic backgrounds in the respective country or state must be considered for comparison or evaluation of the treatment methods and cost-effectiveness.

### 4.3. Geographical and Substantive Differences

While most studies have been conducted in the United Kingdom of Great Britain and Northern Ireland, the public health system in the countries of England, Wales, Scotland, and Northern Ireland must be considered for population care [28,29,31,34,35,47,49]. In direct comparison to the United States (USA) [27,33,37,42], unlike the United Kingdom of Great Britain and Northern Ireland, the USA does not have direct universal coverage or government health protection through health insurance for the entire population. This is in clear contrast to the German health care system [36,43], which finances dental care or oral health care through the German social insurance system in addition to accident, pension, unemployment, and long-term care insurance in the statutory health insurance as well as the private health insurance or guarantees health care including oral health and teeth for the legally insured [36,43]. Accordingly, there is a statutory legal entitlement to comprehensive care with services that are sufficient, appropriate, and economical and do not exceed what is necessary. Although similar, the same conditions are not offered to permanent residents in Australia, so that while medical services are fully subsidized, dental services are only partially subsidized by government insurance [30,39]. To be able to make valid statements about the oral health care, the prevention and restoration measures for ECC in the respective countries, the gross national product (GNP), the care options (type of practices, comprehensive or patchy care), the health protection (health insurance), if existing, individual and group prophylaxis programs (public health care), as well as the access to oral health care would have to be examined when taking the cost-effectiveness into account.

Only one study each on outreach/education/education [27] and sealing [37] were identified; however, the findings are not comparable. The data on pitting and fissure sealing in healthy molars in children at high risk of caries would save approximately USD 25 with a documented follow-up period of five years in the USA [37]. Unfortunately, these data are not comparable due to a lack of evidence, and the statement is therefore not very meaningful regarding cost-effectiveness.

However, regarding the first visit to the dentist, one study was conducted in a large number of children, also in the USA [27], showing that children treated by a dentist for the first time below the age of four years required fewer treatments such as restorations, crowns, pulpotomies, or extractions than children treated by a dentist for the first time when they were over four years old [27]. This fact can also be observed by dentists in individual and group prophylaxis programs in Germany [15]. Lower expenditure demonstrates the cost-effectiveness of getting children used to the dentist and oral care measures as a part of daily personal hygiene at an early age. Additionally, it is encouraging that nowadays in Central European countries, primary prophylaxis is increasingly performed routinely, often in an interdisciplinary way by gynecologists and midwives in cooperation with dentists [15]. In this program, mothers-to-be are already informed and educated about various topics such as sugar consumption and nutrition, oral hygiene, and dental care for their future infants and toddlers [15]. Pre-school prophylaxis then includes prophylaxis at the dentist’s office and in kindergarten through visits by the health department or dentists. The daily habits of brushing teeth with fluoride toothpaste and toothbrush from the first tooth and the regular application of fluoride varnish at the dentist’s visit are introduced at these times and often practiced through play and reward systems [15].

Unfortunately, the different types of treatments reported in the included studies do not allow for a more detailed, concrete assessment of cost-effectiveness, such as with fluoride varnish; although randomized controlled trials have been conducted [18,28,35,36,38,41,49], the statements provide little meaningful results with fluoride varnish application at 98.76 CLP/child in Chile [18] but at 155.74 GBP/child or a combination of prevention and restoration at 250 GBP/child [34,49] in the United Kingdom of Great Britain and Northern Ireland [28]. Although on one hand, in Chile, the fluoride varnish protocol is reported to be more effective and cost-effective in preventing early childhood caries, contradictory results are reported in the United Kingdom of Great Britain and Northern Ireland, such that fluoride varnish application is sufficiently effective, but the effect is only small and the clinical and economic benefits are even questionable [28]. Other studies even documented no difference between the different treatment approaches in terms of clinical outcome [33,49]. No differences in the occurrence or number of toothache episodes or infections due to early carious lesions were also observed over the observation period when comparing the intervention group with the control group, with cost effectiveness slightly in the direction of fluoride varnish application [33,49].

On one hand, it is highly questionable when, based on uncontrolled studies, statements are made that the application of fluoride varnish in one to three sessions in healthy children appears to be cost-effective and therefore the estimated benefit for the prevention of early childhood caries is given [41]. On the other hand, contradictory statements on regular fluoride varnish application in addition to daily tooth brushing under guardian supervision were reported without significant caries preventive effect and even without cost-effectiveness in a study from South Africa [38].

### 4.4. Limitations

This systematic review has several limitations. There is a lack of current prevalence data on the experience of ECC in infants and toddlers and cost-effectiveness studies in numerous countries worldwide. While there are many publications from the United Kingdom of Great Britain and Northern Ireland [28,29,31,33,35,47,49], the United States [27,33,37,42], and Brazil with low to moderate number of publications [32,43,46,48], in the American continent, nine studies [18,27,32,33,37,42,43,46,48]; in the African continent, three studies [38,41,45]; in the Australian continent, two studies [30,39]; and in the Asian continent, only one study [41] were available. In addition to the data on cost-effectiveness in ECC in infants and toddlers from the United Kingdom of Great Britain and Northern Ireland, few relevant data were available in the literature meeting the search criteria of the study to be included in the study. However, because the two continents of Europe and the Americas are reasonably well distributed across the globe, they can at least provide a general estimate of the prevalence of ECC and the importance of cost-effectiveness in this geographic area. It should also be remembered that health data are not generally collected or available for all countries worldwide, clearly affecting the overall estimate of global prevalence. Because the data in the studies were collected in regional or local populations, the results may be only partially representative of the overall situation at the country level. Relatively few studies overall could be included in the systematic review. Although data collection worldwide on cost-effectiveness in public health was initiated in many places about a decade ago, the information is still not extensive and therefore limited. This also means that the statements so far are of little to no significance regarding various preventive as well as restorative therapeutic decision-making processes. The accuracy of the collected and summarized data is also directly dependent on the accuracy of the included data and should therefore be interpreted with caution. Generalization of the data to the respective preventive and restorative therapeutic decisions studied should be made very cautiously, or even avoided, according to the current assessment of the evidence regarding clinical outcomes in cost-effectiveness studies, until the knowledge gaps in the literature are closed.

However, the included studies might show that the prevalence of ECC in infants and toddlers varies widely across geographic areas. More research is needed to monitor oral health status at the individual level (parents or guardians and children), as there is a clear link between healthy oral habits and socioeconomic challenges within societies. This underscores the need for further research on socioeconomic indicators related to socioeconomic, cultural, and ethnic disparities, regardless of the target projection to minority or vulnerable populations. The problem of persistently high caries prevalence can be solved by addressing the needs of parents or guardians in the community with full consideration of the socioeconomic, cultural, as well as ethnic backgrounds. The success of ECC prevention programs depends directly on parents and guardians, but also on doctors and dentists. They are responsible for educating and protecting general and oral health of infants and toddlers. These prevention programs for ECC must be developed with a clear understanding of the individual needs of society and its socioeconomic, cultural, and ethnic circumstances with all decision-makers involved in the health process. Only in this way, will it be possible, together with all decision-makers, to make better treatment decisions in the future, in the sense of a best-practice approach or from a health, economic, and political point of view. Spread of ECC in infants and toddlers must be prevented to avoid the serious consequences of incorrect or inadequate oral and dental hygiene, such as possible damage to the germs of permanent teeth, impaired tooth eruption, developmental disorders of the upper and lower jaws, impaired speech development, insufficient nutritional behavior, dysfunctional chewing, and swallowing ability, but also psychosocial problems. The goal must also be to prevent chewing and swallowing disorders, as well as psychosocial developmental deficits and lack of compliance in future dental visits due to early caries experience.

## 5. Conclusions

The enormously important aspect of public health care ‘cost-effectiveness’ was systematically examined in this study in relation to treatment decisions for ECC in infants and young children, and the following conclusions can be drawn within the limitations of the present study:

1. Statements on the cost-effectiveness of individual therapeutic interventions in the fields of prevention and restoration are difficult due to the heterogeneity in the results or the different methodologies used in the studies included.
2. Despite numerous randomized controlled clinical trials with follow-up periods of often 24 months, the efficacy of individual interventions is sometimes contradictory in terms of clinical outcome.
3. Socioeconomic, cultural, and ethnic differences must be considered when comparing conditions in terms of cost-effectiveness.
4. Studies on cost-effectiveness of therapeutic treatment decisions for early childhood caries have been conducted for about ten years; since then, the number has increased significantly.
5. An important paradigm shift can be observed, away from surgical/restorative approaches and toward increased therapeutic/medical intervention with a focus on prevention.

## Figures and Tables

**Table 1:** Synthesis of the studies included in the systematic review with information on ID no., authors, year of publication, country of study conduct, study design, treatment (intervention), and control group divided into prevention (information/education/education, sealing, fluoride varnish, and silver diamine fluoride (SDF) and atraumatic restorative treatment (ART)) and restoration (filling and crown).

ID	Authors	Year	Country	Study Design	Treatment	Control
Prevention						
Education						
1	Nowak et al. [27]	2014	USA	Retrospective cohort study	Early starters (age at first visit to dentist < 4 years)	Late starters (age at first dental visit > 4 years)
Sealing						
2	Halasa-Rappel et al. [37]	2021	USA	5-year retrospective cohort study	Pitting and fissure sealing (sealed)	No pitting and fissure sealing (not sealed)
Fluoride varnish						
3	Zaror et al. [18]	2020	Chile	Two-year follow-up, triple-blind, randomized, controlled trial	Fluoride varnish	Control (placebo)
4	McMahon et al. [35]	2020	UK	Double-blind, two-arm, randomized, controlled trial	Fluoride varnish application plus treatment-as-usual (TAU) and Childsmile program interventions	TAU and Childsmile program interventions
5	Buckingham & John [29]	2017	UK	Uncontrolled cohort study	Fluoride varnish applications (one, two, and three)	
6	Tickle et al. [28]	2016	UK	Two-arm, randomized, controlled, parallel-group study	Intervention: composite, fluoride varnish 22,600 (ppm), toothbrush, and 50-mL toothpaste at 1450 ppm; and standardized, evidence-based prevention counseling at semiannual intervals for 3 years	The control group received prevention counseling alone
7	Homer et al. [33]	2020	UK	Three-arm, randomized, controlled, parallel-group study	Conventional with best practice prevention (C + P), C + P (e.g., local anesthesia, removal of carious tissue and restoration)	Biological with best practice prevention (B + P): B + P (e.g., partial/no removal and restoration of carious tissue) or best practice prevention alone (PA)
8	Maguire et al. [49]	2020	UK	Multicenter, three-arm, participant-randomized, controlled, parallel-group study	(1) Best practice prevention (local anesthesia, removal of carious tissue, placement of fillings)	(2) Best practice prevention (sealing of caries, selective removal of carious tissue, and fissure sealing) and (3) best practice prevention alone (dietary and toothbrushing recommendations, topical fluoride, and fissure sealing of permanent teeth)
9	Effenberger et al. [45]	2022	South Africa	Multicenter, two-arm, single-blind, cluster-randomized, controlled superiority trial with parallel groups	Fluoride varnish every 3 months and supervised brushing with fluoridated toothpaste	No fluoride varnish, supervised brushing with fluoride toothpaste
10	Nantanee & Sriratanaban [41]	2023	Thailand	Retro and prospective cohort study	Fluoride varnish	
Silver diamine fluoride (SDF) and atraumatic restorative treatment (ART)						
11	Nguyen et al. [39]	2022	Australia	Australian single cohort study	SDF + standard support	SDF—without standard support
12	Tonmukayakul & Arrow [30]	2017	Australia	Pragmatic, randomized, controlled trial	Atraumatic restorative treatment (ART)	Standard care (SC)
13	Bottega et al. [32]	2018	Brazil	Randomized, descriptive, and analytic clinical trial	Papacarie group (caries removal with the chemical–mechanical method—Papacarie gel)	Drill group (caries removal with the traditional method—drilling)
14	Aly et al. [47]	2023	Egypt	Randomized, two-arm, parallel-group, controlled trial (allocation ratio 1:1)	Silver-modified atraumatic restorative treatment (SMART)	Atraumatic restorative treatment (ART)
15	Davis et al. [33]	2020	USA	Retrospective cohort study	Silver diamine fluoride (SDF)	No SDF
16	BaniHani et al. [31]	2018	UK	Retrospective (cost-effectiveness)/prospective cohort study (patient + caregiver acceptance/outcome)	Non-selective removal of hard dentin with/without pulp therapy	Selective removal of solid dentin and the Hall technique
17	Elhennawy et al. [36]	2021	Germany	Two-arm, single-blind, randomized, controlled superiority trial with parallel groups	Selective caries removal (SE)	Stepwise caries removal (SW)
**Restoration**						
Filling						
18	de Moura et al. [46]	2019	Brazil	Randomized controlled trial	Vitro Molar^®^	Ketac Molar^®^
19	Olegário et al. [43]	2017	Brazil	Three-arm, parallel, randomized clinical trial	G1-GC Fumi IX Gold Label 9 (GC Corp)	G2-Vitro Molar (DFL) and G3-Maxxion R (FGM)
20	Olegário et al. [48]	2020	Brazil	Double-blind (participants and evaluators), randomized, three-arm (1:1:1 allocation) clinical trial	G1-GC Fumi IX Gold Label 9 (GC Corp)	G2-Vitro Molar (DFL) and G3-Maxxion R (FGM)
Crown						
21	Schwendicke et al. [43]	2018	Germany	Randomized study	Hall technology (HT)	Non-restorative cavity control (NRCC) and conventional carious tissue removal and restoration (CR)
22	Elamin et al. [45]	2019	Sudan	Randomized clinical trial	Preformed metal crowns (PMCs) placed with conventional techniques (CTs)	PMCs placed by the biological reverberation technique (HT)
23	Schwendicke et al. [47]	2019	UK	Randomized, controlled, split-mouth practice trial	Hall technology (HT)	Conventional removal and restoration of carious tissue (CR)
24	Holsinger et al. [42]	2016	USA	Retrospective cohort study	Zircon crowns	

**Table 2:** Studies included in the systematic review with information on ID number, authors, year of publication, source of publication, site of study conduct, duration of study in months, site of study conduct (practice/clinic), number of participants, age of participants in years, and quality assessment (risk of bias) of the studies. The NIH quality assessment tool rates the observational, cross-sectional, and controlled intervention studies as good, fair (adequate), or poor.

No.	Authors	Year	Source	Country	Duration (Months)	Practice/Clinic	*n* (Participants)	Age (Years)	Quality
1	Nowak et al. [27]	2014	Pediatric Dentistry	USA	96	20 dental care centers	*N* = 42,532 (*n* = 17,040 early starters, first dental visit < 4 years old, *n* = 25,492 late starters, first dental visit > 4 years old).	0 to 7	Good
2	Holsinger et al. [42]	2016	Pediatric Dentistry	USA	6–37, 6–8 (*n* = 14), 14 (*n* = 30)	Pediatric dental practice	18 from 53 children (57 crowns)	2 to 6	Fair
3	Tickle et al. [28]	2016	Health Technology Assessment	Northern Ireland, UK	36	22 NHS dental practices in Northern Ireland and the United Kingdom	1096 from 1248 children	2 to 3	Good
4	Buckingham & John [29]	2017	British Dental Journal	UK	1	Two sites in Southampton and Oxfordshire and three in Portsmouth	458 (589)	4 to 7	Fair
5	Olegário et al. [43]	2017	Journal of Dentistry	Brazil	2, 6, and 12 months	Public schools in the city	150 milk molars in 150 children	4 to 8	Good
6	Tonmukayakul & Arrow [30]	2017	Community Dental and Oral Epidemiology	Australia	12	Real-world practice of public health service	254 children	<5	Fair
7	BaniHani et al. [31]	2018	Caries Research	UK	77	Two dental clinics	Retrospectively *n* = 246 (114 conventional, 132 organic), prospectively *n* = 110	4 to 9	Good
8	Bottega et al. [32]	2018	Scientific Reports	Brazil	1	Municipal schools	24 (12 girls/12 boys), 48 restorations	5.9	Good
9	Schwendicke et al. [44]	2018	Journal of Dentistry	Germany	30	Department of Preventive and Pediatric Dentistry, University Medical Center Greifswald, Germany	142 from 169 children	3 to 8	Good
10	de Moura et al. [46]	2019	Brazilian Oral Research	Brazil	1	Day care centers	243 (728 restorations) included, 1077 preschoolers participated with 21,540 teeth evaluated	2 to 6	Good
11	Elamin et al. [45]	2019	Plos One	Sudan	24	General dental practices	86 children with 109 PMCs (HT) and 78 children with 103 PMCs (CT)	5 to 8	Good
12	Schwendicke et al. [47]	2019	Journal of Dental Research	Scotland, UK	60	17 general dental practices in Scotland in rural, urban, and mixed locations	264 milk molars (132 children)	4 to 9 (10)	Good
13	Davis et al. [33]	2020	The Journal of Clinical Pediatric Dentistry	USA	12	Documentation/patient records of one practice, two private practices	104 (SDF), 250 (non-SDF)	6	Fair
14	Homer et al. [34]	2020	BMC Oral Health	UK	35.5 (min 23, max 36)	General dental practices in England, Scotland, and Wales	1058 from 1144 children	3 to 7	Fair
15	Maguire et al. [49]	2020	Health Technology Assessment	UK	33.8 (23.8–36.7)	72 primary care dental practices	1058 from 1144 children	3 to 7	Fair
16	Nowak et al. [27]	2020	Community Oral Health	UK	24 (6 month interval)	Dental practice, dental outpatient clinics in hospitals	1150 of 1284 children (*n* = 577 FV, *n* = 573 TAU, 10% drop-out).	3.5	Good
17	Olegário et al. [48]	2020	Journal of Dentistry	Brazil	24	27 public schools in the city	150 out of 1200 children evaluated	4 to 8	Good
18	Zaror et al. [18]	2020	Journal of Dentistry	Chile	24	Public rural preschools in areas without access to fluoridated water	275	2 to 3	Fair
19	Elhennawy et al. [36]	2021	Clinical Oral Investigations	Germany	24	Dental Clinic of the Charité—Universitätsmedizin Berlin, Germany	74 children (1 molar/child)	3 to 9	Good
20	Halasa-Rappel et al. [37]	2021	Journal of the American Dental Association	USA	60	Outpatient clinic or operating room	390 children (1884 milk molars)	<6	Fair
21	Effenberger et al. [38]	2022	Community Dental and Oral Epidemiology	South Africa	24	Two schools in the township	513 (from 717)	4 to 8	Fair
22	Nguyen et al. [39]	2022	Australian Dental Journal	Australia	6	Examination of dental services	102 Victorian children	2 to 10	Fair
23	Aly et al. [40]	2023	Journal of Dentistry	Egypt	12	Outpatient Clinic of the Department of Pediatric Dentistry and Public Dental Health	67 (SMART group (*n* = 34, 59 molars) and ART control group (*n* = 33, 60 molars)).	5 to 9	Good
24	Nantanee & Sriratanaban [41]	2023	Community Dental and Oral Epidemiology	Thailand	9–12, 18, 24 and 30	Two randomly selected districts in each of Thailand’s three provinces	460 children	9 to 30 months (19.2 ± 1.7 months)	Good

**Table 3:** Study quality assessment tool NHLBI, NHI; https://www.nhlbi.nih.gov/health-topics/study-quality-assessment-tools; last accessed 12 September 2023.

Authors		1	2	3	4	5	6	7	8	9
Nowak et al. [27]		1	1	1	1	0	1	1	1	1
Holsinger et al. [42]		1	1	1	1	1	NR	1	1	1
Tickle et al. [28]		1	1	1	0	NR	1	1	1	1
Buckingham & John [29]		1	1	1	1	0	NR	1	1	1
Olegário et al. [43]		1	1	1	1	NR	1	1	1	1
Tonmukayakul & Arrow [30]		1	NR	NR	NR	NR	1	1	1	1
BaniHani et al. [31]		1	1	1	1	1	1	1	NR	1
Bottega et al. [32]		1	1	1	1	1	1	1	1	1
Schwendicke et al. [43]		1	1	NR	NR	NR	1	1	1	1
de Moura et al. [46]		1	1	1	1	1	1	0	1	1
Elamin et al. [45]		1	1	1	1	1	NR	1	1	1
Schwendicke et al. [47]		1	1	1	NA	NR	1	0	1	1
Davis et al. [33]		1	1	NA	1	1	1	1	1	1
Homer et al. [34]		1	1	0	NR	NR	1	0	1	1
Maguire et al. [49]		1	1	0	0	0	1	0	1	1
Nowak et al. [27]		1	1	1	1	0	1	1	1	1
Olegário et al. [48]		1	1	1	1	NR	1	1	1	1
Zaror et al. [18]		1	1	1	1	1	1	NR	NR	1
Effenberger et al. [38]		1	1	0	0	1	1	0	0	1
Elhennawy et al. [36]		1	1	0	0	1	1	1	1	1
Halasa-Rappel et al. [37]		1	1	1	1	1	0	1	1	1
Nantanee & Sriratanaban [41]		1	1	1	1	0	1	1	1	1
Nguyen et al. [39]		1	1	1	1	0	1	NA	NA	1
Aly et al. [40]		1	1	0	0	1	1	1	1	1
Legend: NA (not applicable); CD (cannot determine); NR (not reported)										
Grading for CCSS:		0–5 = poor	6–10 = fair	11–14 = good						
Grading for CIS:		0–5 = poor	6–10 = fair	11–14 = good						
10	11	12	13	14	Total	Grading	Quality Assessment			
0	1	NR	1	1	11	Good	For observational and cross-sectional studies			
0	1	NR	0	1	10	Fair	For observational and cross-sectional studies			
NR	1	1	1	1	11	Good	For controlled intervention studies			
1	1	0	1	0	10	Fair	For observational and cross-sectional studies			
1	1	1	1	1	13	Good	For controlled intervention studies			
1	1	0	1	1	9	Fair	For controlled intervention studies			
1	1	0	1	0	11	Good	For observational and cross-sectional studies			
1	1	0	1	1	13	Good	For controlled intervention studies			
1	1	1	1	1	11	Good	For controlled intervention studies			
1	1	1	1	1	13	Good	For controlled intervention studies			
1	1	1	1	0	12	Good	For controlled intervention studies			
1	1	1	1	1	11	Good	For controlled intervention studies			
0	1	0	NA	1	10	Fair	For observational and cross-sectional studies			
1	1	0	1	1	9	Fair	For controlled intervention studies			
1	1	1	1	1	10	Fair	For controlled intervention studies			
1	1	1	1	1	13	Good	For controlled intervention studies			
1	1	1	1	1	13	Good	For controlled intervention studies			
1	1	0	1	0	10	Fair	For controlled intervention studies			
1	1	0	1	0	8	Fair	For controlled intervention studies			
1	1	0	1	1	11	Good	For controlled intervention studies			
0	1	NA	NR	1	10	Fair	For observational and cross-sectional studies			
1	1	1	0	0	11	Good	For observational and cross-sectional studies			
0	1	0	NR	0	7	Fair	For observational and cross-sectional studies			
1	1	0	1	1	11	Good	For controlled intervention studies			

**Table 4:** Studies included in the systematic review with information on ID no., authors, year of publication, source of publication, place of study conduct, cost of treatment (intervention), cost of control group, and clinical outcome.

ID	Authors	Year	Country	Treatment Costs	Control Costs	Clinical Results
Prevention						
Education						
1	Nowak et al. [27]	2014	USA	Early starters: fillings USD 3.11 ± 3.77, crowns 2.28 ± 3.35, pulpotomies USD 1.57 ± 2.66, extractions USD 0.72 ± 1.44, total 7.69 ± 8.61 USD/child/treatment	Late starters: fillings USD 3.96 ± 5.09, crowns USD 3.47 ± 5.13, pulpotomies USD 2.42 ± 4.27, extractions USD 1.41 ± 2.72, total 11.27 ± 12.56 USD/child/treatment	1. Early starters received fewer treatments for restorations, crowns, pulpotomies, and extractions than late starters. 2. Early starters had lower expenditures for treatment procedures than late starters
Sealing						
2	Halasa-Rappel et al. [37]	2021	USA	Pit-and-fissure sealing: 75 USD/molar	No pit-and-fissure sealing: 90 USD/molar	Pit-and-fissure sealing of healthy molars in high-risk children would save USD 25 per molar and result in an additional caries-free molar year over a 5-year follow-up period
Fluoride varnish						
3	Zaror et al. [18]	2020	Chile	Fluoride varnish: 98.76 CLP/child	Control: 98.74 CLP/child	The fluoride varnish protocol is more effective and less expensive in preventing ECC in non-fluoridated areas
4	McMahon et al. [35]	2020	UK	Fluoride varnish plus treatment as usual (TAU) Childsmile program interventions: 32.66 (SD 13.21) GBP/child	TAU Childsmile interventions (Cost: N/A)	An NNT of 21 and cost of GBP 686 to prevent a single worsening of d3mft were calculated. FV is unlikely to be an effective or cost-effective addition to the program
5	Buckingham & John [29]	2017	UK	Fluoride varnish applications (one, two, and three): 71 GBP/child, 88 GBP/child/2 yr	No control	Establishing community fluoride varnish programs requires significant investment, and the long-term benefits in practice are unclear. In addition to fluoridation, dietary improvement is needed to reduce the extent of caries
6	Tickle et al. [28]	2016	UK	The intervention was composite in nature and included a varnish containing 22,600 parts per million (ppm) fluoride, a toothbrush, and a 50-mL tube of toothpaste containing 1450 ppm fluoride; and standardized, evidence-based prevention counseling at semiannual intervals for 3 years: 155.74 GBP/child	The control group received prevention counseling alone: 48.21 GBP/child	No statistically significant effect could be demonstrated for the primary endpoint. Once caries develops, pain is to be expected. There was a statistically significant difference in dmfs in caries-active children in favor of the intervention. Although the intervention was sufficiently effective, the impact was small and the clinical and economic benefits were questionable
7	Homer et al. [34]	2020	UK	Conventional with best practice prevention (C + P), C + P (e.g., local anesthesia, removal of carious tissue and restoration): 250.48 (221.70) GBP/child	Biological with best practice prevention (B + P; e.g., partial/no removal and restoration of carious tissue): 231.28 (214.47) GBP/child, or best practice prevention alone (PA): 211.32 (257.28) GBP/child	At the thresholds (mean cost), B + P has the highest probability of being considered cost-effective. Beyond the willingness-to-pay thresholds considered, the probability of B + P being considered cost-effective never exceeded 75%
8	Maguire et al. [49]	2020	UK	(1) Best practice prevention (local anesthesia, removal of carious tissue, placement of fillings): 250.48 (221.70) GBP/child	(2) Best practice prevention (sealing of caries, selective removal of carious tissue, and fissure sealing): 231.28 (214.47) GBP/child, and (3) best practice prevention alone (dietary and toothbrushing counseling, topical fluoride, and fissure sealing) of permanent teeth: 211.32 (257.28) GBP/child	There was no evidence of an overall difference between the three treatment approaches in the incidence or number of episodes of toothache or infection or both, during the follow-up period
9	Effenberger et al. [38]	2022	South Africa	Fluoride varnish every 3 months and supervised brushing with fluoridated toothpaste: 1667 (SD 1055) ZAR/child	No fluoride varnish, supervised tooth brushing with fluoridated toothpaste: ZAR 950 (SD 943)	Regular application of fluoride varnish in addition to daily tooth brushing under supervision had no significant caries preventive effect and was not cost-effective
10	Nantanee & Sriratanaban [41]	2023	Thailand	Fluoride varnish: 50.30 ± 24.14 THB per visit/child	No control	The fluoride varnish application program in one to three sessions during visits in healthy children appears to be cost-effective and shows estimated net benefits of interventions to prevent dental caries. These results suggest that children between the ages of 9 and 30 months should have at least three visits of the fluoride varnish application program during child care visits
Silver diamine fluoride (SDF) and atraumatic restorative treatment (ART)						
11	Nguyen et al. [39]	2022	Australia	Children receiving SDF + standard care: AUD 409.90 (SD 36.24); SDF protocol intervention: AUD 3.48 per child	Children receiving SDF—no standard care; SDF protocol intervention: AUD 3.48 per child	The SDF protocol intervention is a cost-effective option for the treatment of dental caries in young children
12	Tonmukayakul & Arrow [30]	2017	Australia	Atraumatic restorative treatment (ART): AUD 1085.51 (includes general anesthesia (GA))	Standard care (SC): AUD 1403.28 (incl. GA)	Children in the ART-based group received more dental services than those in the SC group. The ART-based approach appears to be a worthwhile intervention, as it resulted in fewer referred cases and allowed more treatments to be delivered with cost savings
13	Bottega et al. [32]	2018	Brazil	Papacarie group (caries removal with the chemical–mechanical method—Papacarie gel): 0.91 BRL/procedure	Drill group (caries removal with the traditional method—drilling): 1.37 BRL/procedure	Papacarie offers an excellent cost advantage for minimally invasive removal of carious tissue and is a viable alternative for public health care
14	Aly et al. [40]	2023	Egypt	Silver-modified atraumatic restorative treatment (SMART): 67 EGP (USD 2.17)/average cost per restoration of the two study arms	Atraumatic restorative treatment (ART): 61 EGP (USD 1.97)/average cost per restoration of the two study arms	The use of SMART technology could change paradigms in caries management. Because it is a patient-friendly and cost-effective approach, it could be used as a superior treatment option in treating young children, people with behavioral and medical problems, and to promote access to oral care for the disadvantaged
15	Davis et al. [33]	2020	USA	Silver diamine fluoride (SDF): 619.72 USD (SD 563.51)/child/year (number of visits/dental treatments and expenditures)	No SDF: 958.04 USD (SD 824.65)/child/year (number of visits/dental treatments and expenditures)	SDF can result in cost savings when used as an adjunct to, rather than a complete replacement for, restorative treatment in young children
16	BaniHani et al. [31]	2018	UK	Non-selective removal of hard dentin with/without pulp therapy: 168.68 GBP/child	Selective removal of solid dentin and Hall technique: 78.97 GBP/child	Although both approaches achieved similar successful results, the biological approach, which consisted mainly of the Hall technique, was associated with lower treatment costs. Both approaches were positively received by the children and caregivers
17	Elhennawy et al. [36]	2021	Germany	Selective caries removal (SE): 68.4 EUR (20.1) (initial cost)/1 molar/child; total treatment cost after 24 months: EUR 85 (74); total cost after 24 months: EUR 106 (90)	Stepwise caries removal (SW): 132.9 EUR (18.3) (initial cost)/1 molar/child; total treatment cost after 24 months: EUR 141 (44); total cost after 24 months: EUR 176 (51)	In deciduous molars with clearly defined deep carious lesions, SE was less expensive and similarly effective to SW. For cost and applicability reasons, SW may have to be indicated restrictively, e.g., only for very deep lesions (>3/4 dentin extension)
**Restoration**						
Filling						
18	de Moura et al. [46]	2019	Brazil	Vitro Molar^®^: low-cost GIC (exact cost: N/A)	Ketac Molar^®^: high-viscosity GIC (exact cost: N/A)	The success rate for restorations with low-cost GIC (Vitro Molar^®^) was particularly high after six months. However, one year later, restorations performed with high-viscosity GIC (Ketac Molar^®^) in primary second molars with small or medium cavities were more successful than those indicated for ART with low-cost GIC
19	Olegário et al. [43]	2017	Brazil	G1-GC Fuji IX Gold Label 9 (GC Corp): 55 BRL/package	G2-Vitro Molar (DFL): 25 BRL/package and G3-Maxxion R (FGM): 9.4 BRL/package.	The low-cost GICs perform worse than GC Fuji Gold Label 9 in occlusal ART restorations in deciduous molars
20	Olegário et al. [48]	2020	Brazil	G1-GC Fuji IX Gold Label 9 (GC Corp): BRL 4.66 (0.13) (baseline); BRL 5.32 (0.28); BRL 5.88 (0.38) (2-year total cost).	G2-Vitro Molar (DFL): BRL 3.65 (0.11) (baseline); BRL 5.225 (0.30) (2-year total cost) and G3-Maxxion R (FGM): BRL 3.37 (0.08) (baseline); BRL 4.84 (0.26) (2-year total cost)	The use of GC Gold Label 9 shows a higher survival rate compared to Maxxion R and Vitro Molar for occlusal ART restorations in primary molars, i.e., low-cost GICs perform worse than GC Gold Label 9 for occlusal ART restorations in primary molars
Crown						
21	Schwendicke et al. [44]	2018	Germany	Hall technique (HT): 66 (62–71) EUR/child (1 molar)	Non-restorative cavity control (NRCC): 296 (274–318) EUR/child (1 molar) and conventional removal and restoration of carious tissue (CR): 83 (73–92) EUR/child (1 molar)	HT was more cost-effective than CR or NRCC in treating cavitated caries lesions in deciduous molars and resulted in better dental health outcomes at lower cost
22	Elamin et al. [45]	2019	Sudan	Preformed metal crowns (PMCs) placed using conventional techniques (CT): 7.81 SDG/unit	PMCs placed by biological reverberation techniques (HT): 2.45 SDG/unit	PMCs placed using the Hall technique or conventional techniques have excellent survival rates in disadvantaged communities. Extremely cost-effective in terms of materials, labor, and time, HT is a successful and cost-effective public health intervention for carious deciduous molars in communities and developing countries
23	Schwendicke et al. [47]	2019	UK	Hall technique (HT)—intervention: 24 (23–25) GBP/child/molar (base case analysis); 32.26 (30.83–33.98) GBP/child/molar	Conventional removal and restoration of carious tissue (CR): 29 (25–34) GBP/child/molar (base case analysis); 48.91 (34.40–68.74) GBP/child/molar	Based on a long-term practice-based study, HT was more cost-effective than CR because HT was maintained longer and fewer complications occurred at a lower cost
24	Holsinger et al. [42]	2016	USA	Zircon crowns; zircon crown: USD 23.48 (approximate cost of crown mold), USD 28.38 (total estimated cost of materials/treatment)	No control—cost indicated for: resin-veneered stainless-steel crown: USD 18.70 (approximate crown mold cost), USD 24.13 (estimated total material/treatment cost); strip crown: USD 6.18 (approximate crown mold cost), USD 21.83 (estimated total material/treatment cost)	Zirconia crowns are clinically acceptable restorations in the primary maxillary anterior dentition

## Data Availability

Data sharing is not applicable to this article as no new data were created or analyzed in this study.

## Supplementary Materials

The following supporting information can be downloaded at: https://www.mdpi.com/article/10.3390/medicina59101865/s1, Figure S1: PRISMA 2020 checklist; Table S1: Excluded studies; Figure S2: PRISMA flow diagram.
