# Supplementary material for: Cost-Effectiveness of Treatment Decisions for Early Childhood Caries in Infants and Toddlers: A Systematic Review
Source: Medicina (Kaunas). 2023 Oct 20;59(10):1865. doi: 10.3390/medicina59101865 (PMC10608526; doi:10.3390/medicina59101865)
Supplement: Supplementary file 1 [file medicina-59-01865-s001.zip › Table S1. Excluded studies.pdf]

ID	Autoren	Quelle	Grund Exlusion
1	van Ligten et al.	Community Dentistry and Oral Epidemiology 2023 [Article in press]	NR
2	Ono et al.	Community Dentistry and Oral Epidemiology 2023; 51: 2228-235	NR
3	Tabatabai & Kjellberg	European Journal of Orthodontics 2023 [Article in press]	Review
4	Egil & Yaylali	Journal of Public Health Dentistry 2023; 831: 43-50	NR
5	Lin et al.	JDR Clinical and Translational Research 2023 [Article in Press]	NR
6	Ruff et al.	JAMA Network Open 2023; 62: E2255458	NR
7	Surtie et al.	Cleft Palate-Craniofacial Journal 2023 [Article in Press]	NR
8	Bhatt et al.	Dental Research Journal 2023; 20:1 18	NR
9	Khairinisa et al.	BMC Oral Health 2023; 23(1): 172	NR
10	Kateeb et al.	BMC Oral Health 2023; 23(1): 152	NR
11	Azimi et al.	International Journal of Paediatric Dentistry 2023; 33(3): 234-245	NR
12	Chiba et al.	Caries Research 2023; 57(1): 43-51	NR
13	Ariyavutikul et al.	International Journal of Paediatric Dentistry 2023; 33(2): 113-123	NR
14	Wu et al.	Clinical Oral Investigations 2023; 27(2): 773-785	NR
15	Manchanda et al.	Archives of Oral Biology 2023; 146: 105607	NR
16	Oliveira et al.	Acta Odontologica Scandinavica 2023; 81(3): 216-226	NR
17	Folayan et al.	International Journal of Paediatric Dentistry 2023; 33(1): 74-81	NR
18	Tsai et al.	International Journal of Paediatric Dentistry 2023; 33(1): 1-11	NR
19	Gupta et al.	Community Dentistry and Oral Epidemiology 2022; 50(5): 430-436	NR
20	Wang et al.	BMJ Open 2022; 12(9): e061601	NR
21	Zolnikov et al.	Journal of Public Health Dentistry 2022; 82:4 365-371	NR
22	Da Silva et al.	Journal Canadian Dental Association 2022; 88: m9	NR
23	Mehta et al.	Clinical and Experimental Allergy 2022; 52:8 1053	NR
24	Yusuf et al.	Archives of Disease in Childhood 2022; 107 (Supplement 2): A338	NR
25	Wajahat et al.	Journal of Taibah University Medical Sciences 2022; 17:3 408-414	NR

26	Sanghvi et al.	Community Dentistry and Oral Epidemiology 2022 [Article in Press]	NR
27	Lucas-Rincon et al.	Community Dental Health 2022; 39:2 86-91	NR
28	Victory et al.	Applied Health Economics and Health Policy 2022; 20(3): 431-445	NR
29	Anopa et al.	JDR Clinical and Translational Research 2022 [Article in Press]	NR
30	Herndon et al.	Journal of Public Health Dentistry 2022; 82:2 176-185	NR
31	Okuji et al.	MedRxiv 2022 [Article in Press]	NR
32	Migas et al.	International Journal of Environmental Research and Public Health 2022; 19(4): 2183	NR
33	Meyer et al.	Journal of Prevention 2022; 43:1 111-123	NR
34	Karthiga et al.	Journal of Pharmaceutical Negative Results 2022; 13 1353-1358	NR
35	Jivraj et al.	The Journal of Evidence-Based Dental Practice 2022; 22:1 101662	Fallbericht
36	Prabhu et al.	Journal of the Indian Society of Pedodontics and Preventive Dentistry 2022;	NR
37	Ganesh et al.	Caries Research 2022; 56(4): 399-406	NR
38	Nizar et al.	Clinical and Experimental Dental Research 2022; 8(6): 1523-1532	NR
39	Kimmie-Dhansay et al.	BMC Oral Health 2022; 22(1): 183	Review
40	Kimmie-Dhansay et al.	BMC Oral Health 2022; 22(1): 32	Review
41	Panchanadikar et al.	Journal of Indian Society of Pedodontics and Preventive Dentistry 2022; 437-444	NR
42	Boustedt et al.	European Archives of Paediatric Dentistry 2022; 23(5): 829-833	NR
43	Chai et al.	Journal of Dentistry 2022; 125: 104250	NR
44	Liao et al.	International Journal of Paediatric Dentistry 2022; 32(5): 627-638	NR
45	Tsuchiya et al.	Community Dentistry and Oral Epidemiology 2022; 50(4): 300-310	NR
46	Chevvuri et al.	Indian Journal of Dental Research 2022; 33(3): 297-300	NR
47	Peedikayil et al.	Journal of Indian Society of Pedodontics and Preventive Dentistry 2022; 40(3): 317-323	NR
48	Cho et al.	Caries Research 2022; 56(2): 129-137	NR
49	Vered et al.	Quintessence International 2022; 53(6): 546-555	NR

50	Tantikalchan & Mitrakul	European Archives of Paediatric Dentistry 2022; (3): 437-447	NR
51	Park & Choi	International Dental Journal 2022; 72(3): 392-398	NR
52	Yawary & Hegde	International Dental Journal 2022; 73(3): 322-330	Protokoll
53	Graesser et al.	International Dental Journal 2022; 72(3): 381-391	NR
54	Lara et al.	International Journal of Paediatric Dentistry2022; 32(3): 334-343	NR
55	Tsai & Lawrence	International Journal of Paediatric Dentistry 2022; 32(3): 352-366	NR
56	Zulekha et al.	Journal of Indian Society of Pedodontics and Preventive Dentistry 2022; 40(2): 159-164	NR
57	Panchanadikar et al.	The Journal of Clinical Pediatric Dentistry 2022; 46(2): 75-85	NR
58	Agarwal et al.	The Journal of Clinical Pediatric Dentistry 2022; 46(2): 125-131	NR
59	Pereira et al.	Clinical Oral Investigations 2022; 26(2): 1605-1612	NR
60	Nogueira et al.	Journal of Dentistry for Children 2022; 89(1): 11-17	NR
61	Gudipaneni et al.	The Journal of Clinical Pediatric Dentistry 2022; 46(1): 35-43	NR
62	Aguirre et al.	European Journal of Paediatric Dentistry 2022; 23(1): 15-20	NR
63	Agouropoulos et al.	International Journal of Paediatric Dentistry 2022; 32(1): 82-898	NR
64	Guclu et al.	International Journal of Clinical Practice 2021; 75(12): e14888	NR
65	Liu et al.	Journal of stomatology 2021; 39:6 703-708	NR
66	Avenetti et al.	Journal of Dentistry for Children Chicago, Ill 2021; 88(3): 187-195	NR
67	Schwendicke et al.	Journal of Dentistry; 2021: 103751	NR
68	Haq et al.	Fluoride 2021; 54(3): 210-218	NR
69	Aripirala et al.	Journal of the Indian Society of Pedodontics and Preventive Dentistry 2021; 39(3): 303-309	NR
70	Taylor et al.	BMC Oral Health 2021; 21:1 318	Review
71	Alshehri et al.	Australian Health Review; 2021	NR
72	Da Silva et al.	BMC Oral Health 2021; 21(1): 256	Protokoll
73	Rajput et al.	Special Care in Dentistry 2021; 41:3 358-366	NR
74	Alhareky	Saudi Journal of Medicine and Medical Sciences 2021; 9(2): 113-117	Review
75	Bianchi et al.	BMC Oral Health 2021; 21(1): 167	Protokoll

76	Cronin et al.	BMC Oral Health 2021; 21(1): 158	NR
77	Folayan et al.	BMC Oral Health 2021; 21(1): 126	NR
78	Tonmukayakul et al.	Australian Dental Journal 2021; 66(Supplement 1): S63-S70	NR
79	Abduazimova et al.	International Journal of Pharmaceutical Research 2021; 13(1): 3752-3757	NR
80	Lalloo et al.	PLoS ONE 2021; 16(1): e0244927	NR
81	Mendes et al.	F1000 Research 2021; 9: 650	Protokoll
82	Sitthisettapong et al.	Frontiers in Public Health 2021; 9 664541	NR
83	Oliviera et al.	Brazilian Oral Research 2021; 35: e126	NR
84	Vaghela et al.	Journal of Contemporary Dental Practice 2021; 22(12): 1462-1470	NR
85	Gou et al.	BMC Oral Health 2021; 21(1): 664	NR
86	Deghatipour et al.	BMC Oral Health 2021; 21(1): 637	NR
87	Zhou et al.	BMC Oral Health 2021; 21(1): 572	NR
88	Andrew et al.	BMC Oral Health 2021; 21(1): 521	Review
89	Faheem et al.	BMC Oral Health 2021; 21(1): 445	NR
90	Folayan et al.	BMC Oral Health 2021; 21(1): 415	NR
91	Alade et al.	BMC Oral Health 2021; 21(1): 73	NR
92	Ladeira et al.	Community Dentistry and Oral Epidemiology 2021; 49(6) 602-608	NR
93	Zheng et al.	BMC Oral Health 2021; 21(1): 181	NR
94	Leelataweewud et al.	BMC Oral Health 2021; 21(1): 64	NR
95	Culler et al.	Journal of Public Health Dentistry 2021; 81(1): 29-41	NR
96	Almoudi et al.	Journal of Clinical Pediatric Dentistry 2021; 45(5): 330-336	NR
97	McGivern et al.	Journal of the American Dental Association 2021; 152(11): 936	NR
98	Ruan et al.	Archives of Oral Biology 2021; 131: 105220	NR
99	Abirami et al.	Caries Research 2021; 55(5): 554-562	NR
100	Kaur et al.	Journal of Indian Society of Pedodontics and Preventive Dentistry 2021; 39(4): 403-408	NR

101	Charak et al.	Journal of Indian Society of Pedodontics and Preventive Dentistry 2021; 39(4): 379-383	NR
102	Anderson et al.	European Archives of Paediatric Dentistry 2021; 22(5): 947-957	NR
103	Hernandez et al.	European Archives of Paediatric Dentistry 2021; 22(5): 833-842	NR
104	Jordan et al.	Journal of Dentistry for Children 2021; 88(3): 156-163	NR
105	Thomas et al.	Journal of Public Health Dentistry 2021; 81(3): 224-231	NR
106	Chen et al.	Caries Research 2021; 55(4): 310-321	NR
107	Smith et al.	Community Dentistry and Oral Epidemiology 2021; 49(3): 284-290	NR
108	Al-Sane et al.	European Archives of Paediatric Dentistry 2021; 22(3): 449-458	NR
109	BaniHani et al.	European Archives of Paediatric Dentistry 2021; 22(3): 433-439	NR
110	Pereira et al.	European Archives of Paediatric Dentistry 2021; 22(3): 399-408	NR
111	Bayram et al.	Clinical Oral Investigations 2021; 25(5): 2867-2876	NR
112	Wattanarat et al.	Clinical Oral Investigations 2021; 25(5): 2891-2903	NR
113	Sowmya et al.	Indian Journal of Dental Research 2021; 32(2): 147-152	NR
114	Abedizadeh et al.	Journal of Indian Society of Pedodontics and Preventive Dentistry 2021; 39(2): 196-201	NR
115	Elelmi et al.	European Archives of Paediatric Dentistry 2021; 22(2): 235-240	NR
116	Mohamed et al.	Australian Dental Journal 2021; 66(S1): S27-S36	NR
117	Colvara et al.	Community Dentistry and Oral Epidemiology 2021; 49(1): 10-16	Review
118	Severino et al.	European Journal of Paediatric Dentistry 2021; 22(3): 189-198	NR
119	Buckeridge et al.	International Journal of Paediatric Dentistry 2021; 31(1): 115-121	NR
120	Machado et al.	Trials 2020; 21(1): 874	Protokoll
121	Ekstrand et al.	International Journal of Circumpolar Health 2020; 79(1): 1804260	NR
122	Simpson et al.	Evidence-Based Dentistry 2020; 21:4 128-129	NR
123	Singh et al.	BMC Oral Health 2020; 20:1 320	NR
124	Asif & Emg	International Journal of Research in Pharmaceutical Sciences 2020; 11:4 5956-5959	NR
125	Lakshmanan et al.	Indian Journal of Forensic Medicine and Toxicology 2020; 14:4 5864-5870	NR
126	Sankar et al.	Indian Journal of Forensic Medicine and Toxicology 2020; 14:4 5964-5972	NR
127	Roth el al.	Journal of Graduate Medical Education 2020; 12:5 571-577	NR
128	Aarthi et al.	European Journal of Molecular and Clinical Medicine 2020; 7(2): 6468-6473	NR

129	Warren et al.	Journal of Public Health Dentistry 2020; 80(3): 254-256	NR
130	Richards	Evidence-Based Dentistry 2020; 21(3): 100-101	NR
131	Ludwig et al.	Journal of Public Health Dentistry 2020; 80(Supplement 2): S122-S125	NR
132	Freeman et al.	Community Dentistry and Oral Epidemiology 2020; 48:4 328-337	NR
133	Vu et al.	Pharmaceutics 2020; 12(7): 1-16	NR
134	Freiberg et al.	International Journal of Environmental Research and Public Health 2020; 17(8): 2672	NR
135	Meyer et al.	JDR Clinical and Translational Research 2020; 5(2): 146-155	NR
136	El-Yousfi et al.	BMC Oral Health 2020; 20(1): 69	NR
137	Anopaet al.	Evidence-Based Dentistry 2020; 21(1): 5-7	NR
138	Kularatna et al.	Health and Quality of Life Outcomes 2020; 18(1): 43	NR
139	Cavalcante et al.	International Journal of Environmental Research and Public Health 2020; 17(3): 1076	NR
140	Pine et al.	Journal of Dental Research 2020; 99(2): 168-174	NR
141	Priyadarshini & Gurunathan	International Journal of Research in Pharmaceutical Sciences 2020; 11(4): 2201-2213	NR
142	Medina-Solis et al.	Medicine United States 2020; 99(7): e19092	NR
143	Umeda et al.	Brazilian Oral Research 2020; 34: e017	NR
144	Nagarajappa et al.	Roczniki Panstwowego Zakladu Higieny 2020; 71(1): 113-122	NR
145	Folayan et al.	BMC Oral Health 2020; 20(1): 336	NR
146	Gao et al.	Journal of Dentistry 2020; 103: 103522	NR
147	Lee et al.	BMC Oral Health 2020; 20(1): 285	NR
148	Piwat et al.	Caries Research 2020; 54(45082): 491-501	NR
149	Hariyani et al.	Community Dentistry and Oral Epidemiology 2020; 48(6): 561-569	NR
150	Muthu et al.	Community Dentistry and Oral Epidemiology 2020; 48(6): 471-479	NR
151	Wagner et al.	Clinical Oral Investigations 2020; 24(12): 4313-4324	NR
152	Mei et al.	Journal of Dentistry 2020; 102: 103479	NR
153	Zhu et al.	International Journal of Dental Hygiene 2020; 18(4): 352-361	NR
154	Folayan et al.	International Journal of Paediatric Dentistry 2020; 30(6): 798-804	NR
155	Kubota et al.	Oral Health & Preventive Dentistry 2020; 18(1): 973-980	NR

156	Lotto et al.	Journal of Dentistry 2020; 101:103456	NR
157	Ibrahim & Nourallah	Clinical and Experimental Dental Research 2020; 6(5): 537-543	NR
158	Muraleedhar et al.	Community Dentistry and Oral Epidemiology 2020; 48(5): 379-386	NR
159	Lamba et al.	Clinical Oral Investigations 2020; 24(10): 3467-3475	NR
160	Jordan et al.	Journal of Public Health Dentistry 2020; 80(4): 271-277	NR
161	Kyoon-Achan et al.	Journal of Public Health Dentistry 2020; 80(3): 208-216	NR
162	Nembhwani & Winnier	International Journal of Paediatric Dentistry 2020; 30(5): 619-625	NR
163	Azadani et al.	Journal of the American Dental Association 2020; 151(8): 568-575	NR
164	Mabangkhru et al.	Journal of Dentistry 2020; 99: 103375	NR
165	Arrow & Forrest	Community Dentistry and Oral Epidemiology 2020; 48(4): 349-356	NR
166	Sun	Acta Odontologica Scandinavica 2020; 78(5): 352-257	NR
167	Rajab & Abdullah	Oral Health and Preventive Dentistry 2020; 18(3): 571-582	NR
168	Jiang et al.	Journal of Dentistry 2020; 87:103349	NR
169	Arrow & Forrest	Australian Dental Journal 2020; 65(2): 158-167	NR
170	Kim & Koo	Journal of Dental Research 2020; 99(6): 597-603	NR
171	Chandhru et al.	Journal of Indian Society of Pedodontics and Preventive Dentistry 2020; 38(2): 152-157	NR
172	Barjatya et al.	Journal of Indian Society of Pedodontics and Preventive Dentistry 2020; 38(2): 98-103	NR
173	Naidu & Nunn	Oral Health and Preventive Dentistry 2020; 18(2): 245-252	NR
174	Duangthip et al.	International Dental Journal 2020; 70(2): 100-107	NR
175	Blue et al.	BMC Oral Health 2020; 20(1): 90	NR
176	Folayan et al.	BMC Oral Health 2020; 20(1): 54	NR
177	Nagarajappa et al.	European Archives of Paediatric Dentistry 2020; 21(1): 67-74	NR
178	Folayan et al.	BMC Oral Health 2020; 20(1): 8	NR
179	Nahas & Sfeir	Journal of Contemporary Dental Practice 2020; 21(9): 1012-1017	NR
180	Norrie & Pharand	Canadian Journal of Dental Hygiene 2020; 548(2): 68-74	NR

181	Olczak-Kowalczyk et al.	Oral Health and Preventive Dentistry 2020; 18(4): 833-842	NR
182	Yokoi et al.	Acta Odontologica Scandinavica 2020; 1-6	NR
183	Meriç et al.	European Journal of Paediatric Dentistry 2020; 21(1): 13-17	NR
184	Da Silva et al.	Journal of Dentistry for Children 2020; 87(1): 12-17	NR
185	Samuel et al.	Journal of Public Health Dentistry 2020; 80(1): 51-60	NR
186	Weintraub et al.	Journal of Public Health Dentistry 2019; 79(4): 298-306	NR
187	Kroon et al.	Community Dentistry and Oral Epidemiology 2019; 47:6 470-476	NR
188	Dahlberg et al.	Journal of pediatric Health Care 2019; 33:6 702-710	NR
189	Huang et al.	JDR Clinical and Translational Research 2019; 4(4): 378-387	NR
190	Fernando et al.	JDR Clinical and Translational Research 2019; 4(4): 333-341	NR
191	Johhnson et al.	Journal of Public Health Dentistry 2019; 79(3): 215-221	NR
192	Arora et al.	Cochrane Database of Systematic Reviews 2019; 8: CD012595	Review
193	Fraihat et al.	International Journal of Environmental Research and Public Health 2019; 16(15): 2668	Review
194	Al Baghdadi et al.	Pediatrics 2019; 144:2	NR
195	Rogers et al.	BMC Oral Health 2019; 19(1): 132	Review
196	Fernandez-Barrera et al.	Medicine 2019; 98(30): e16634	Protokoll
197	Broden et al.	Acta Odontologica Scandinavica 2019; 77(4): 275-281	NR
198	Park et al.	Clinical Oral Investigations 2019; 23(5): 2383-2387	NR
199	Chen	Hu li za zhi The Journal of Nursing 2019; 66:1 4	NR
200	Da Silva et al.	BMC Oral Health 2019; 19:1 6	Protokoll
201	Elzembely et al.	Pediatric Blood and Cancer 2019; 66(1): e27421	NR
202	Harrita et al.	Drug Invention Today 2019; 11(7): 1564-1567	NR
203	Folayan et al.	BMC Oral Health 2019; 20(1): 1	NR
204	Sharma et al.	Journal of Indian Society of Pedodontics and Preventive Dentistry 2019; 37(4): 339-344	NR
205	Faustino-Silva & Figueiredo	Clinical Oral Investigations 2019; 23(10): 3721-3729	NR

206	Dharmani et al.	Indian Journal of Dental Research 2019; 30(5): 742-746	NR
207	Kelly et al.	Journal of Public Health Dentistry 2019; 79(3): 264-270	NR
208	Purnima et al.	Journal of Indian Society of Pedodontics and Preventive Dentistry 2019; 37(3) 232-236	NR
209	Turton et al.	BMC Oral Health 2019; 19(1): 107	NR
210	Rubin et al.	Journal of Public Health Dentistry 2019; 79(2): 116-123	NR
211	Angelopoulou et al.	Journal of Public Health Dentistry 2019; 79(2): 102-108	NR
212	Bartosova et al.	BMC Oral Health 2019; 19(1): 33	NR
213	Winter et al.	Clinical Oral Investigations 2019; 23(1): 187-197	NR
214	Hurley et al.	BMC Oral Health 2019; 19(1): 13	NR
215	Colombo et al.	European Journal of Paediatric Dentistry 2019; 20(4): 267-273	NR
216	Ferrazzano et al.	European Journal of Paediatric Dentistry 2019; 20(3): 214-218	NR
217	Muthu et al.	Oral Health and Preventive Dentistry 2019; 17(3): 277-282	NR
218	Meyer et al.	BMC Oral Health 2018; 18(1): 215	NR
219	Schwendicke et al.	[In Process] BMJ Open 2018; 8(12): e022952	Protokoll
220	Sibley	Journal of Pediatric Health Care 2018; 32:6 620-626	NR
221	Lee et al.	Health Services Research 2018; 53(5): 3592-3616	NR
222	Burgette & Quinonez	JDR Clinical and Translational Research 2018; 3(4): 336-345	NR
223	Wang & Chen	Value in Health 2018; 21(Supplement 2): S58	NR
224	Hastie et al.	American Journal of Hematology 2018; 93(9): E24-E25	NR
225	Estai et al.	Australian Health Review 2018; 42(5): 482-490	Review
226	Persaud et al.	BMJ Open 2018; 8(8): e020941	Protokoll
227	Hu et al.	European Academy of Paediatric Dentistry 2018; 19(4): 221-227	NR
228	Chen et al.	Trials 2018; 19(1): 352	Protokoll
229	Folayan et al.	The New Bioethics 2018; 24(2): 135-149	NR
230	Trudnak et al.	Maternal and Child Health Journal 2018; 22(7): 998-1007	NR
231	Phantumvanit et al.	Community Dentistry and Oral Epidemiology 2018; 46(3): 280-287	NR
232	Friedman et al.	Journal Canadian Dental Association 2018; 84: i5	NR

233	Ruff & Niederman	BMJ Open 2018; 8(4): e022646	Protokoll
234	Ladewig et al.	Expert Review of Pharmacoeconomics and Outcomes Research 2018; 18(2): 127-134	Review
235	Goldman et al.	Journal of Dentistry 2018; 70: 80-86	NR
236	Khouja & Smith	Journal of Public Health Dentistry 2018; 78(2): 118-126	NR
237	Marino et al.	BMC Oral Health 2018; 18(1): 24	NR
238	Lakshmi et al.	Journal of Clinical and Diagnostic Research 2018; 12(2): ZC09-ZC13	NR
239	Schwendicke et al.	Community Dentistry and Oral Epidemiology 2018; 46(1): 8-16	NR
240	Zhang et al.	BMC Oral Health 2018; 18(1): 203	NR
241	Neves et al.	Archives of Oral Biology 2018; 96: 155-161	NR
242	Xiao et al.	Journal of Dental Research 2018; 97(13): 1468-1476	NR
243	Canares et al.	General Dentistry 2018; 66(6): 24-28	NR
244	Gavic et al.	International Journal of Paediatric Dentistry 2018; 28(6): 616-623	NR
245	Evans et al.	Community Dentistry and Oral Epidemiology 2018; 46(5): 518-525	Protokoll
246	Seminario et al.	Journal of Dentistry for Children 2018; 85: 93-101	NR
247	Jain et al.	Indian Journal of Dental Research 141; 29(5): 568-574	NR
248	Ismail et al.	BMC Oral Health 2018; 18(1):126	NR
249	Chandna et al.	Journal of Indian Society of Pedodontics and Preventive Dentistry 2018; 36(3): 290-295	NR
250	Shackleton et al.	Community Dentistry and Oral Epidemiology 2018; 46(3): 288-296	NR
251	Govindaraju et al.	Indian Journal of Dental Research 2018; 29(3): 313-316	NR
252	Campagna et al.	Pediatric Dentistry 2018; 40(3): 210-214	NR
253	George et al.	Journal of Indian Society of Pedodontics and Preventive Dentistry 2018; 36(2): 130-134	NR
254	Owen et al.	Australian Dental Journal 2018; 63(1): 72-80	NR
255	Muñoz-Millán et al.	Community Dentistry and Oral Epidemiology 2018; 46(1): 63-69	NR
256	Ozler et al.	Oral Health and Preventive Dentistry 2018; 16(5): 467-472	NR
257	Gürlek et al.	Oral Health and Preventive Dentistry 2018; 16(5): 457-465	NR
258	Gao et al.	Journal of Clinical Pediatric Dentistry 2018; 42(5): 367-372	NR

259	Rataj-Kulmacz et al.	Oral Health and Preventive Dentistry 2018; 16(4): 363-368	NR
260	Villavicencio et al.	Journal of Applied Oral Science 2018; 26: e20170318	NR
261	Igic et al.	European Journal of Paediatric Dentistry 2018; 19(2): 161-164	NR
262	Nguyen et al.	Journal of Clinical Pediatric Dentistry 2018; 42(3): 173-181	NR
263	Ugolini et al.	Oral Health and Preventive Dentistry 2018; 16(1): 87-92	NR
264	Costa et al.	Caries Research 2018; 51(6): 582-589	NR
265	Edelstein	Dent Clin North Am 2017; 613: 589-606	NR
266	Johnson et al.	Community Dentistry and Oral Epidemiology 2017; 45:6 522-528	NR
267	Levey et al.	Trials 2017; 18(1): 515	Review
268	Crystal et al.	Pediatric Dentistry 2017; 39(5): 135-145	NR
269	Dhar et al.	Pediatric Dentistry 2017; 39(5): 146-159	NR
270	Gupta et al.	Community Dental Health 2017; 34(3): 131-136	Review
271	Hansen et al.	Pediatric Dentistry 2017; 39(4): 304-307	NR
272	Jabeen & Umbreen	Journal of the Liaquat University of Medical and Health Sciences 2017; 16(3): 170-174	NR
273	Foster et al.	JDR Clinical and Translational Research 2017; 2(3): 287-294	NR
274	Bindi et al.	British Dental Journal 2017; 222(10): 809-817	NR
275	Flood et al.	Academic Pediatrics 2017; 17(3): 316-322	NR
276	Ganapule et al.	Blood Coagulation and Fibrinolysis 2017; 28(2): 171-175	Review
277	Baric & Gabric	Acta Stomatologica Croatica 2017; 51(1): 86	Fallbericht
278	Chao et al.	Paediatric Anaesthesia 2017; 27(1): 98-105	NR
279	Chestnutt et al.	Health Technology Assessment 2017; 21: 21	NR
280	Goldman et al.	Caries Research 2017; 51(5): 489-499	NR
281	Tsai et al.	Journal of the Chinese Medical Association 2016; 79(8): 456-463	NR
282	Li et al.	BMC Oral Health 2017; 17(1): 144	NR
283	Javed et al.	Journal of Investigative and Clinical Dentistry 2017; 8(4)	Review
284	Colombo et al.	Archives of Oral Biology 2017; 83: 282-288	NR
285	Chen et al.	Archives of Oral Biology 2017; 83: 174-180	NR

286	Beena et al.	Journal of Indian Society of Pedodontics and Preventive Dentistry 2017; 35(4): 296-300	NR
287	Faizunisa et al.	Journal of Indian Society of Pedodontics and Preventive Dentistry 2017; 35(4): 291-295	NR
288	Anderson et al.	Journal of Dentistry 2017; 65: 83-88	NR
289	Meyer et al.	Community Dentistry and Oral Epidemiology 2017; 45(5): 442-448	NR
290	Heaton et al.	Journal of Public Health Dentistry 2017; 77(4): 350-359	NR
291	Mitrakul et al.	European Archives of Paediatric Dentistry 2017; 18(4): 251-261	NR
292	Wigen et al.	International Journal of Dental Hygiene 2017; 15(3): 249-255	NR
293	Valdez et al.	BMC Oral Health 2017; 17(1): 115	NR
294	Bhat et al.	Journal of Indian Society of Pedodontics and Preventive Dentistry 2017; 35(3): 193-197	NR
295	Neves et al.	Clinical Oral Investigations 2017; 21(6): 2053-2061	NR
296	Hernandez et al.	Journal of the American Dental Association 2017; 148(6): 392-398	NR
297	Arrow& Klobas	Australian Dental Journal 2017; 62(2): 200-207	NR
298	Chaffee et al.	Community Dentistry and Oral Epidemiology 2017; 45(3): 216-224	NR
299	Collado et al.	Medicina Oral, Patologia Oral y Cirugia Bucal 2017; 22(3): e333-e341	NR
300	Priyadarshini et al.	Journal of Indian Society of Pedodontics and Preventive Dentistry 2017; 35(2): 110-114	NR
301	Kowash et al.	European Archives of Paediatric Dentistry 2017; 18(2): 97-103	NR
302	Thompson et al.	Pediatric Dentistry 2017; 39(2): 124-129	NR
303	Piva et al.	Brazilian Dental Journal 2017; 28(2): 241-248	NR
304	Kakanur et al.	Indian Journal of Dental Research 2017; 28(1): 27-32	NR
305	Jiang	Oral Health and Preventive Dentistry 2017; 15(1): 89-97	NR
306	Wagner & Heinrich-Weltzien	Clinical Oral Investigations 2017; 21(1): 225-235	NR
307	Griffin & Griffin	Journal of Evidence Based Dental Practice 2016; 162: 133-5	NR
308	O'Keefe et al.	Community Dentistry and Oral Epidemiology 2016; 44:6 515-522	NR
309	Mathu-Muju & Kennedy	Pediatric Dentistry 2016; 38(5): 46-53	NR
310	De Silva et al.	Cochrane Database of Systematic Reviews 2016; 9: CD009837	Review
311	Ramanath et al.	Research Journal of Pharmaceutical, Biological and Chemical Sciences 2016; 7(5): 319-327	NR

312	Bruen et al.	Journal of the American Dental Association 2016; 147(9): 702-708	NR
313	Olegario et al.	BMC Oral Health 2016; 17(1): 34	NR
314	Bergstrom et al.	Community Dental Health 2016; 33(2): 138-144	NR
315	Richard	Evidence-Based Dentistry 2016; 17(2): 35-37	NR
316	Baek et al.	International Dental Journal 2016; 66(3): 136-143	NR
317	Lawrence et al.	British Dental Journal 2016; 220(10): 545-547	NR
318	Gray-Burrows et al.	Implementation Science 2016; 11: 61	NR
319	Wilberg et al.	Supportive Care in Cancer 2016; 24(4): 1497-1506	NR
320	Ribeiro et al.	Ciencia & Saude Coletiva 2016; 21(4): 1217-1226	NR
321	Mendes et al.	Trials 2016; 17(1): 69	Protokoll
322	Gao et al.	BMC Oral Health 2016; 16: 12	Review
323	Pretty & Ekstrand	European Archives of Paediatric Dentistry 2016; 17(1): 13-25	Review
324	Riggs et al.	BMC Pregnancy and Childbirth 2016; 16(1): 12	NR
325	Soni et al.	Pravara Medical Review 2016; 8(1): 27-29	Review
326	Hesse et al.	Trials 2016; 17(1): 169	Protokoll
327	Griffin et al.	Health Affairs 2016; 35(12): 2233-2240	NR
328	Bonetti & Clarkson	Caries Research 2016; 50(Supplement 1): 45-49	NR
329	Neidell et al.	Caries Research 2016; 50(Supplement 1): 78-82	NR
330	Astvaldsdottir et al.	Caries Research 2016; 50(4): 383-393	Review
331	Naidu et al.	BMC Oral Health 2016; 16(1): 128	NR
332	Fan et al.	BMC Oral Health 2016: 16(1): 98	NR
333	Castro et al.	Journal of Dentistry for Children 2016; 83(3): 125-131	NR
334	Memarpour et al.	Caries Research 2016; 55(5): 433-442	NR
335	Dabiri et al.	International Dental Journal 2016; 66(4): 221-228	NR
336	Edelstein et al.	Pediatric Dentistry 2016; 38(4): 325-330	NR
337	Colombo et al.	Archives of Oral Biology 2016; 67: 22-27	NR
338	Ng & Fida	Journal of Evidence-Based Dental Practice 2016; 16: 20-33	NR
339	Arrow & Klobas	Australian Dental Journal 2016; 61(2): 227-235	NR

340	Bezerra et al.	Caries Research 2016; 50(3): 279-287	NR
341	Mothupi et al.	Journal of Dentistry for Children 2016; 83(2): 83-87	NR
342	Turton et al.	European Archives of Paediatric Dentistry 2016; 17(2): 97-105	NR
343	Rodríguez et al.	Journal of Dental Research 2016; 95(4): 402-407	NR
344	Custodio-Lumsden et al.	Journal of Public Health Dentistry 2016; 76(2): 136-142	NR
345	Arrow	Caries Research 2016; 50(1): 1-8	NR
346	Arrow	Community Dentistry and Oral Epidemiology 2016; 44(1): 1-10	NR
347	Paglia et al.	European Journal of Paediatric Dentistry 2016; 17(2): 93-99	NR
348	Schroth et al.	Journal of Public Health Dentistry 2016; 76(3): 206-212	NR
349	Gopal et al.	Oral Health and Preventive Dentistry 2016; 14(3): 267-273	NR
350	Cunha-Cruz et al.	Trials 2015; 16: 278	Protokoll
351	Fyfe et al.	New Zealand Medical Journal 2015; 128(1427): 38-46	NR
352	Wright et al.	BMC Oral Health 2015; 15: 160	NR
353	Goyal et al.	The Journal of Clinical Pediatric Dentistry 2015; 39(2): 109-112	NR
354	Pine et al.	Trials 2015; 16(1): 505	Protokoll
355	Jaeger et al.	Journal of Emergency Medicine 2015; 49(5): 729-739	NR
356	Jackson	Primary Dental Journal 2015; 4(4): 46-51	NR
357	Stewart et al.	Primary Dental Journal 2015; 4(4): 67-73	NR
358	Chu et al.	Trials 2015; 16(1): 426	Protokoll
359	Gao et al.	Trials 2015; 16(1): 416	Protokoll
360	Tonmukayakul et al.	Contemporary Clinical Trials 2015; 44: 36-41	Protokoll
361	Lalloo et al.	BMC Oral Health 2015; 15: 99	NR
362	Anopa et al.	PLoS ONE 2015; 10(8): e0136211	NR
363	Ekstrand 6 Qvist	International Journal of Paediatric Dentistry 2015; 25(4): 255-266	NR
364	Casamassimo & Seale	Journal of Dental Education 2015; 79(6): 644-652	NR

365	Alsharif et al.	Journal of Public Health Dentistry 2015; 75(3): 202-209	NR
366	Schwendicke et al.	Journal of Dentistry 2015; 43(6): 647-655	NR
367	Tinanoff	Pediatric Dentistry 2015; 37(3): 198-199	NR
368	Douglass & Clark	Pediatric Dentistry 2015; 37(3): 266-274	NR
369	Compton	Pediatric Dentistry 2015; 37(3): 288-293	NR
370	Edelstein et al.	Journal of the American Dental Association 2015; 146(4): 224-232	NR
371	Mejare et al.	PLoS ONE 2015; 10(2): e0117537	Review
372	Schwendicke	Journal of Dental Research 2015; 94(2): 272-280	NR
373	Conquest et al.	International Dental Journal 2015; 65(1): 32-38	NR
374	Schwendicke et al.	Trials 2015; 16(1): 11	Protokoll
375	Folayan et al.	BMC Oral Health 2015; 15(1): 72	NR
376	Arrow & Klobas	Community Dentistry and Oral Epidemiology 2015; 43(6): 511-520	NR
377	Koh et al.	Community Dentistry and Oral Epidemiology 2015; 43(6): 560-568	NR
378	Samnaliev et al.	Journal of Public Health Dentistry 2015; 75(1): 24-33	NR
379	Ghazal et al.	Journal of Public Health Dentistry 2015; 75(1): 42-48	NR
380	Qiu et al.	BMC Oral Health 2015; 15(1): 144	NR
381	Saraithong et al.	Clinical Oral Investigations 2015; 19(8): 1955-1964	NR
382	Arrow & Klobas	Australian Dental Journal 2015; 60(3): 375-381	NR
383	Romanos et al.	Caries Research 2015; 49(4): 425-433	NR
384	EzEldeen et al.	European Archives of Paediatric Dentistry 2015; 16(4): 333-340	NR
385	Kneist et al.	European Archives of Paediatric Dentistry 2015; 16(4): 365-370	NR
386	Ghazal et al.	Community Dentistry and Oral Epidemiology 2015; 43(4): 366-374	NR
387	Baggio et al.	BMC Oral Health 2015; 15(1): 82	NR
388	Nakayama & Mori	Journal of Public Health Dentistry 2015; 75(2): 157-162	NR
389	Nicol et al.	Australian Dental Journal 2015; 60(1): 73-79	NR
390	Abbaso‡lu et al.	Caries Research 2015; 49(1): 70-77	NR
391	Tannure et al.	Journal of Clinical Pediatric Dentistry 2015; 39(5): 410-414	Fallbericht

392	Mattos-Silveira et al.	Trials 2014; 15: 448	Protokoll
393	Quissell et al.	Contemp Clin Trials 2014; 372: 242-51	NR
394	Bansal et al.	Indian Journal of Dental Research 2014; 25(6): 777-782	NR
395	Zaliskyy et al.	Value in Health 2014; 17(7): A613	NR
396	Zhang & Yang	Value in Health 2014; 17(7): A781-A782	NR
397	Johnson et al.	Australian Dental Journal 2014; 59(3): 366-371	NR
398	Kranz et al.	Journal of Dental Research 2014; 93(7): 633-638	NR
399	Batliner et al.	Trials 2014; 15(1): 125	NR
400	Larson	Northwest Dentistry 2014; 93(2): 35-38	NR
401	Berg	Journal of the California Dental Association 2014; 42(7): 442-447	NR
402	Liu et al.	BMC Oral Health 2014; 14: 54	NR
403	Fisher-Owens	Pediatric Dentistry 2014; 36(2): 115-120	NR
404	Vermaire et al.	Caries Research 2014; 48(3): 244-253	NR
405	Kowash	Applied Clinical Research, Clinical Trials and Regulatory Affairs 2014; 1:2	Review
406	Tam	Journal of Investigative Medicine 2014; 62(1): 247	NR
407	Agouropoulos et al.	Journal of Dentistry 2014; 42(10): 1277-1283	NR
408	Anandakrishna et al.	Indian Journal of Dental Research 2014; 25(5): 602-606	NR
409	Mathur et al.	Indian Journal of Dental Research 2014; 25(5): 559-566	NR
410	Batliner et al.	Journal of Public Health Dentistry 2014; 74(4): 317-325	NR
411	Chi et al.	Journal of Public Health Dentistry 2014; 74(4): 261-265	NR
412	Nicol et al.	BMC Oral Health 2014; 14(1): 69	NR
413	Quinonez et al.	BMC Oral Health 2014; 14(1): 33	NR
414	Pesaressi et al.	BMC Oral Health 2014; 14(1): 17	NR
415	Han et al.	Community Dentistry and Oral Epidemiology 2014; 42(1): 70-78	NR
416	Wulaerhan et al.	BMC Oral Health 2014; 14(1): 136	NR
417	Anitha et al.	Journal of Indian Society of Pedodontics and Preventive Dentistry 2014; 32(3): 220-224	NR

418	Ramos-Jorge et al.	Community Dentistry and Oral Epidemiology 2014; 42(4): 311-322	NR
419	Cuadros Fernández et al.	European Journal of Paediatric Dentistry 2014; 15(1): 229-233	Fallbericht
420	Aljafari et al.	Community Dental Health 2014; 31(2): 75-79	NR
421	Congiu et al.	Journal of Public Health Dentistry 2014; 74(2): 147-152	NR
422	El Batawi	European Archives of Paediatric Dentistry 2014; 15(3): 183-189	NR
423	Kivistö et al.	European Archives of Paediatric Dentistry 2014; 15(3): 197-202	NR
424	Masumo et al.	Acta Odontologica Scandinavica 2014; 72(4): 312-320	NR
425	Bücher et al.	Clinical Oral Investigations 2014; 18(3): 775-782	NR
426	Klinke et al.	Caries Research 2014; 48(1): 24-31	NR
427	Aminabadi et al.	Caries Research 2014; 48(1): 3-12	NR
428	Arrow et al.	BMC Public Health 2013; 13: 245	NR
429	Meihubers	New South Wales Public Health Bulletin 2013; 24(3): 128-130	NR
430	Hutchings et al.	Trials 2013; 14(1): 147	NR
431	Khandelwal et al.	BMJ Case Reports 2013; 2013: bcr2013010029	Fallbericht
432	Monse et al.	BMC Public Health 2013; 13: 256	NR
433	Pukallus et al.	BMJ Open 2013; 3:5 Article Number: 002579	NR
434	Ribeiro et al.	International Journal of Paediatric Dentistry 2013; 23(3): 225-234	NR
435	Hendrix et al.	Journal of Public Health Dentistry 2013; 73(4): 297-303	NR
436	Banoczy et al.	Acta Medica Academica 2013; 42(2): 156-167	NR
437	Innes et al.	BMC Oral Health 2013; 13: 25	Protokoll
438	Raj et al.	BMC Oral Health 2013; 13: 67	NR
439	Prasai Dixit et al.	BMC Oral Health 2013; 13: 20	NR
440	Curtis et al.	Australian Dental Journal 2011; 561: 48-55	NR
441	Maxim et al.	Revista medico-chirurgicala a Societatii de Medici si Naturalisti din Iasi 2010; 1143: 866-9	NR
442	Warren et al.	Value Health 2010; 136: 750-60	NR
443	Freeman & Oliver	Brritish Dental Journal 2009; 20612: 619-25	NR
444	Milnes	Journal of the Canadian Dental Association 2003; 695: 298-302	NR

445	Farsi	Odonto-stomatologie Tropicale 1999; 2286: 27-32	NR
446	Horowitz	Community Dentistry Oral Epidemioly 1998; 261(Suppl): 91-5	NR
