# Supplementary figures and images for: Cost-Effectiveness of Treatment Decisions for Early Childhood Caries in Infants and Toddlers: A Systematic Review
Source: Medicina (Kaunas). 2023 Oct 20;59(10):1865. doi: 10.3390/medicina59101865 (PMC10608526; doi:10.3390/medicina59101865)

Figure S2. PRISMA flow diagram

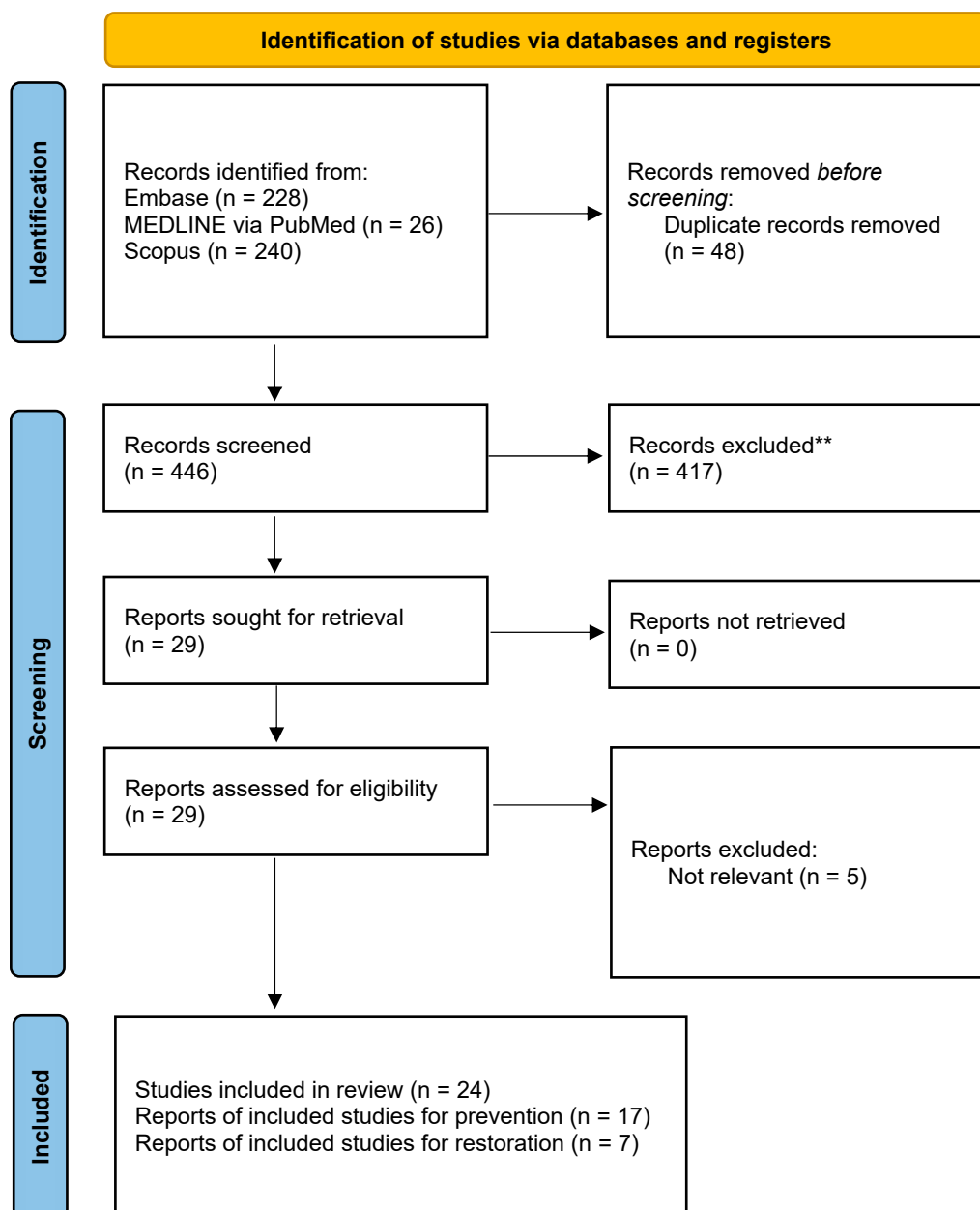

Supplement: Supplementary file 1 [file medicina-59-01865-s001.zip › Figure S2. PRISMA flow diagram.pdf]
